# The WHO priority list of antibiotic-resistant bacteria: challenges and opportunities for next-generation antimicrobial development

**DOI:** 10.3389/fphar.2026.1699987

**Published:** 2026-04-10

**Authors:** Emad M. Abdallah, Abdulrahman Mohammed Alhudhaibi, Mahmoud Dahab, Abdullah Al Noman, Pranab Dev Sharma, Tarek H. Taha, Muhammad Nawaz

**Affiliations:** 1 Department of Biology, College of Science, Qassim University, Buraydah, Saudi Arabia; 2 Department of Biology, College of Science, Imam Mohammad Ibn Saud Islamic University (IMSIU), Riyadh, Saudi Arabia; 3 Faculty of Pharmacy, University of Malaya, Wilayah PersekutuanKuala Lumpur, Malaysia; 4 Department of Chemistry and Biochemistry, Kennesaw State University, Kennesaw, GA, United States; 5 School of Pharmacy, BRAC University, Dhaka, Bangladesh; 6 Biotechnology program, Department of Mathematics and Natural Science, BRAC University, Dhaka, Bangladesh; 7 Institute of Microbiology, University of Veterinary and Animal Sciences, Lahore, Pakistan

**Keywords:** antibiotic resistance, antibiotics, drug discovery, health, infectious disease, novel antimicrobials, WHO priority pathogens

## Abstract

Antimicrobial resistance (AMR) remains one of the most serious global threats to public health, driven by the rapid emergence and dissemination of multidrug-resistant bacterial pathogens that compromise existing antibiotic therapies. In response, the World Health Organization (WHO) has defined priority lists of antibiotic-resistant bacteria to guide research, innovation, and drug development efforts. This narrative review synthesizes current knowledge on the molecular mechanisms underlying resistance in WHO-priority pathogens, including reduced membrane permeability, efflux pump overexpression, enzymatic drug inactivation, target modification, biofilm formation, and horizontal gene transfer. Beyond mechanistic insights, we critically evaluate the therapeutic limitations of conventional antibiotics, the failure of traditional discovery pipelines, and the growing clinical and economic burden of resistant infections. Emerging strategies, including artificial intelligence-assisted drug discovery, phage therapy, antimicrobial peptides, CRISPR-based systems, resistance-modifying combinations, and natural product-derived compounds and plant compounds, are assessed with emphasis on pharmacological feasibility, translational challenges, and clinical relevance. Particular attention is given to issues of delivery, toxicity, dosing optimization, resistance emergence, regulatory barriers, and real-world implementation. Finally, we highlight the central role of antimicrobial stewardship, surveillance, and a One Health framework integrating human, animal, and environmental sectors in mitigating resistance and sustaining therapeutic effectiveness. Collectively, this review underscores that addressing WHO-priority pathogens will require integrated, multidisciplinary strategies that bridge molecular biology, pharmacology, clinical translation, and public health.

## Introduction

Concerningly, the increase of antimicrobial resistance (AMR) has outpaced the discovery of new antibiotics. This means that we are approching a post-antibiotic age, where common illnesses could once again become untreatable (Abdallah et al., 2023). According to 2021 estimates from the Global Burden of Disease (GBD) study, AMR was associated with 4.71 million deaths worldwide. Of these, 1.14 million were a direct consequence of AMR. Current trajectories suggest a continued escalation of this threat through 2050. Analysis of the last 3 decades shows mortality rates from AMR vary considerably based on age and location. By 2050, South Asia, along with Latin America and the Caribbean, are projected to be the super-regions experiencing the most severe all-age mortality rates due to AMR (Naghavi et al., 2024). Current projections indicate that, if left unaddressed, AMR will be responsible for ten million deaths globally by 2050 (Murray et al., 2022).

In 2017, the WHO published a priority list classifying pathogen into medium, high, and critical tiers as a guidance for research, innovation and development efforts addressing AMR (WHO,2017). The classification is based on mortality burden, prevalence of resistance, healthcare impact, and unmet therapeutic need. Carbapenem-resistant pathogens such as *Acinetobacter baumannii* (Moraxellaceae), *Pseudomonas aeruginosa* (Pseudomonadaceae), and members of the family Enterobacteriaceae (e.g., *Klebsiella pneumoniae, Enterobacter* spp.) are associated with severe or life-threatening infections in hospitalized patients, are classified as critical priority in WHO classification list. The high and medium classifications include methicillin-resistant *Staphylococcus aureus* (MRSA), fluoroquinolone-resistant *Salmonella* (Enterobacteriaceae)and *Neisseria gonorrhoeae* (Neisseriaceae), and penicillin-resistant *Streptococcus pneumoniae* (Streptococcaceae), all of which are considered as threaten global public health and require urgent and sustained research and development (R&D) to avoid a post-antibiotic era (WHO, 2017). Seven years later, the WHO published the 2024 Bacterial Priority Pathogens List (BPPL) reflecting updates in response to the growing global burden of AMR. Rifampicin-resistant *Mycobacterium tuberculosis* (Mycobacteriaceae) and third-generation cephalosporin-resistant Enterobacterales are now major priorities, highlighting their global burden. *Pseudomonas aeruginosa* is demoted to high priority, fluoroquinolone-resistant *Shigella* is up, and macrolide-resistant Group A and B are elevated in priority. The WHO also included *Streptococci* to medium priority category. The five pathogen–antibiotic resistance combinations in 2017 WHO classification list, including clarithromycin-resistant *Helicobacter pylori* (Helicobacteraceae), were deleted. The list now comprises 15 pathogen families, using transmissibility, treatability, and R&D pipeline bottlenecks to prioritize AMR research and public health policies worldwide (WHO, 2024). Moreover, WHO BPPL must be interpreted within a One Health continuum linking hospital, agriculture, environment, and climate-driven ecological shifts.

Antibiotics are a double-edged sword. The father of modern antibiotics, Sir Alexander Fleming, warned that “the use of antimicrobials can, and will, lead to resistance.” This warning is now a reality, as the revolutionary potential of antibiotics is being weakened by widespread abuse in human health, agriculture, and veterinary medicine (Caneschi et al., 2023). The period from 1940 to 1990 represented a golden age of antibiotic discovery and development. However, since 1990, the pipeline has diminished significantly, characterized by a declining number of approvals per decade and a complete absence of novel antibiotic classes with distinct chemical structures (Renwick and Mossialos, 2018). The selective pressure resulting from the misuse and overuse of antimicrobials in healthcare, agriculture, and veterinary practice drives microbial populations to develop resistance through adaptation. Consequently, this escalating resistance threatens to render common infections and minor injuries life-threatening once again (Ahmed et al., 2024). Moreover, antibiotic pollution is a critical environmental driver of AMR. Key factors exacerbating this crisis include climate change, pesticide and heavy metal contamination (which co-select for resistance), microplastics that act as hubs for gene transfer, and ecological disturbances that reduce microbial diversity (Abdallah, 2025). From a clinical pharmacology perspective, the greatest short-term progress against WHO-priority Gram-negative pathogens will likely come from strategies that restore intracellular antibiotic activity rather than replace antibiotics. This includes optimising β-lactam/β-lactamase inhibitor pairs, using rational combinations to overcome permeability and efflux barriers, and adding anti-biofilm agents for device-related infections (Harris et al., 2015). By contrast, CRISPR-based antimicrobials and many nanotechnology approaches remain promising but face major challenges in delivery, safety, manufacturing, and regulatory approval, placing them in the mid-to long-term horizon. Phage therapy sits in between: clinical benefits are reported in some refractory infections, yet wider use requires clearer PK/PD data, harmonised regulation, and scalable production. Natural products are currently most realistic as adjunct therapies (resistance-modifying or anti-virulence) unless strong standardisation and human exposure data support development as standalone drugs.

A critical research gap persists in translating this mechanistic knowledge into innovative and effective therapeutic strategies capable of overcoming these defenses. Therefore, a comprehensive analysis is required that not only consolidates the current knowledge of resistance mechanisms in WHO priority pathogens but also critically evaluates the most promising non-traditional strategies, from artificial intelligence-driven drug discovery, phage therapy, and the repurposing of natural compounds, that may offer viable solutions to outpacing resistance (Aggarwal et al., 2024; Guo et al., 2024; Rahman et al., 2022). Accordingly, this narrative review is structured around three interconnected axes: (i) the molecular mechanisms driving antibiotic resistance in WHO-priority pathogens, (ii) the therapeutic limitations and clinical gaps associated with conventional antibiotics, and (iii) emerging and innovative strategies for next-generation antimicrobial development. This framework enables a coherent integration of mechanistic insights with translational and public health perspectives. Throughout this review, molecular resistance mechanisms are interpreted as determinants of drug exposure, dosing strategy, and therapeutic index, thereby directly linking microbiological resistance processes to rational antimicrobial design and clinical pharmacology.

## Methodology

This narrative review brings together current knowledge on WHO-priority antibiotic-resistant pathogens and emerging therapeutic strategies, with emphasis on linking molecular resistance mechanisms to therapeutic barriers and pharmacological solutions. A narrative approach was intentionally adopted because the objective was integrative and conceptual rather than quantitative, aiming to connect mechanistic biology, drug development, and clinical pharmacology—an analytical scope not suited to systematic aggregation of homogeneous studies.

Literature searches were performed in PubMed, Web of Science, Scopus, and Google Scholar for publications from January 2010 to September 2024, supplemented by authoritative reports from the World Health Organization (WHO) and the Centres for Disease Control and Prevention (CDC). Search terms included combinations of “antimicrobial resistance,” “WHO priority pathogens,” “novel antimicrobials,” “drug discovery,” and pathogen-specific keywords. Eligible sources comprised English-language primary research articles, systematic or narrative reviews, and official institutional reports directly addressing WHO-priority bacterial pathogens, resistance mechanisms, or emerging therapeutic strategies. Editorials, conference abstracts, non-peer-reviewed sources, and studies not directly relevant to WHO-listed pathogens were excluded.

Evidence was synthesized thematically rather than quantitatively. Studies were prioritized according to scientific quality, clinical relevance, and their contribution to linking resistance mechanisms with therapeutic implications and pharmacological innovation. The review was structured around a predefined conceptual framework connecting resistance mechanisms to therapeutic limitations and corresponding pharmacological strategies (resistance mechanism → therapeutic limitation → pharmacological solution).

For comparative evaluation, major therapeutic approaches were further assessed based on (i) strength of supporting evidence (human clinical data, animal studies, *in vitro* evidence, or early proof-of-concept) and (ii) translational readiness (near-term, mid-term, or early/high-risk). For each approach, we summarize likely clinical positioning, key development barriers—including delivery, toxicity, scalability, regulatory considerations, and cost—and the anticipated pathway toward clinical implementation.

### Antibiotic resistance mechanisms in WHO priority pathogens

**FIGURE 1:**
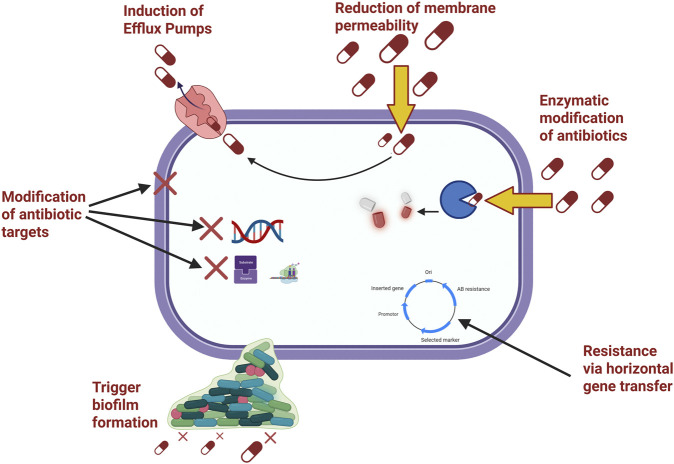
Major mechanisms of antibiotic resistance in WHO priority pathogens. Legend: Schematic representation of the principal cellular mechanisms contributing to bacterial antibiotic resistance. These include reduced membrane permeability limiting antibiotic entry, active efflux pump induction promoting drug extrusion, enzymatic modification or degradation of antibiotics, structural modification of antibiotic targets, acquisition of resistance determinants via horizontal gene transfer, and biofilm formation that enhances collective tolerance. Together, these mechanisms reduce intracellular drug accumulation, impair antibiotic–target interactions, and facilitate the persistence and dissemination of resistant phenotypes.

**FIGURE 2:**
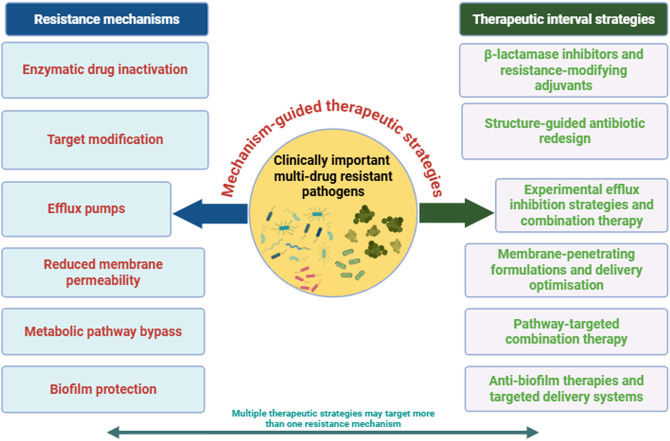
Mechanism-guided therapeutic strategies targeting major antibiotic resistance determinants in clinically important multidrug-resistant pathogens. Legend: Overview of the principal resistance mechanisms in clinically important multidrug-resistant pathogens, including enzymatic drug inactivation, target modification, active efflux, reduced membrane permeability, metabolic bypass, and biofilm protection, and their corresponding mechanism-guided therapeutic strategies. The schematic integrates rational antibiotic redesign, resistance-modifying adjuvants, efflux inhibition, optimized drug delivery systems, pathway-targeted combinations, and anti-biofilm approaches aligned to counter defined resistance determinants, often requiring integrated multi-target interventions (Created with BioRender.com).

Antibiotic resistance in infections caused by WHO-priority pathogenic bacteria is driven by various molecular processes that compromise the effectiveness of commonly used antimicrobials (Tacconelli et al., 2018). The main resistance mechanisms include reduced membrane permeability, increased efflux pump activity, enzymatic modification of antibiotics, alteration of antibiotic targets, and biofilm formation (Figure 1). As summarised in Table 1, resistance across WHO-priority pathogens converges on a limited set of recurring mechanistic themes, such as enzymatic drug inactivation, permeability restriction, efflux-mediated exclusion, and target modification, which collectively shape current antimicrobial design priorities; the full gene-level determinants and supporting references are provided in Supplementary Table S1. Moreover, a mechanistic understanding of resistance architecture is essential for rational therapeutic innovation; accordingly, Figure 2 presents an integrated overview linking dominant resistance pathways in clinically significant multidrug-resistant pathogens to mechanism-guided intervention strategies.

**TABLE 1:** Mechanistic resistance patterns among WHO-priority pathogens and implications for antimicrobial development.^a^

WHO priority level	Representative pathogens	Dominant resistance mechanisms	Implications for antimicrobial development
Critical priority	*Acinetobacter baumannii*, *Pseudomonas aeruginosa*, carbapenem-resistant Enterobacterales	Carbapenemase production, multidrug efflux systems, reduced outer-membrane permeability	Development of β-lactamase-stable agents, efflux-resistant scaffolds, permeability-enhancing combinations, and optimized PK exposure strategies
High priority	*Enterococcus faecium*, *Staphylococcus aureus*, *Helicobacter pylori*, *Salmonella* spp., *Campylobacter* spp., *Neisseria gonorrhoeae*	Target modification, β-lactam resistance, fluoroquinolone resistance, adaptive multidrug resistance	Need for novel targets, anti-virulence strategies, improved dosing optimisation, and resistance-guided therapy selection
Medium priority	*Streptococcus pneumoniae*, *Haemophilus influenzae*, *Shigella* spp.	Altered penicillin-binding proteins, macrolide resistance, emerging multidrug resistance	Importance of dosing optimisation, surveillance-guided therapy, vaccine-linked control, and preservation of existing antibiotic classes

^a^ Mechanistic categories are summarised from the gene-level resistance determinants compiled in Supplementary Table S1 and the WHO, priority pathogen framework (WHO, 2024). The supplementary table provides the full gene-level dataset and primary references supporting these mechanistic groupings (Supplementary Table S1).

### Reduced membrane permeability

In general, a fundamental antibiotic-resistance strategy in Gram-negative bacteria is the reduction of outer-membrane permeability, which frequently contributes to multidrug resistance when combined with efflux activation or enzymatic drug inactivation, as reflected in the broader resistance patterns summarised in Table 1. The reduction in membrane permeability primarily occurs through a decrease in functional entry channels within the outer membrane. This is facilitated by the downregulation, inactivation, or structural alteration of porin proteins, which normally allow passive diffusion of antibiotics into the cell (Masi et al., 2017; Maher and Hassan, 2023). In *Klebsiella pneumoniae*, inactivation or loss of the major outer-membrane porins OmpK35 and OmpK36 significantly reduces permeability to third-generation cephalosporins and carbapenems, contributing to high-level β-lactam resistance (Rocker et al., 2020; Tsai et al., 2011). Similarly, *Escherichia coli* downregulates OmpF and OmpC to limit β-lactam influx (Alzayn et al., 2021). Other examples include *Acinetobacter* baumannii, which modifies CarO and OmpA porins to reduce carbapenem susceptibility, and *Pseudomonas aeruginosa*, which suppresses the OprD porin to block imipenem entry and confer high-level resistance (Uppalapati et al., 2020; Sionov and Steinberg, 2022).

### Efflux pumps

Efflux pumps play a central role in bacterial antimicrobial resistance by reducing intracellular drug concentrations to sub-lethal levels, thereby facilitating survival under antibiotic pressure. In Gram-negative bacteria, multidrug efflux systems confer broad cross-resistance to structurally unrelated antibiotics and biocides, contributing to multidrug-resistant and extensively drug-resistant phenotypes. Importantly, efflux rarely acts in isolation; rather, it synergises with target mutations, enzymatic drug inactivation, and reduced membrane permeability, amplifying resistance levels and expanding the mutant selection window (Nikaido and Pagès, 2012). From a clinical and drug-development perspective, efflux-mediated resistance is particularly problematic because even modest increases in minimum inhibitory concentrations (MICs) can translate into therapeutic failure, while routine susceptibility testing often fails to capture the dynamic and inducible nature of efflux activity. Consequently, efflux pumps remain a persistent barrier to sustainable antimicrobial therapy and a critical target for next-generation resistance-mitigation strategies (Blair et al., 2015).

In Enterobacterales, the AcrAB-TolC efflux system is a major contributor to resistance against important drug classes like fluoroquinolones and cephalosporins (Weston et al., 2018). In *A. baumannii,* resistance to carbapenems is frequently mediated by overexpression of AdeABC and AdeIJK efflux systems (Xu et al., 2019; Roy et al., 2022). *P. aeruginosa* utilize multiple efflux pumps (Table 1), most notably MexAB-OprM efflux, to confer resistance to carbapenem antibiotics (Elshafiee et al., 2022). In *E. coli*, the overexpression of *acrA, acrB, tolC, mexA*, and *mexB* efflux pump genes was found to be associated with reduced susceptibility to carbapenem and other antimicrobial agents (Elshafiee et al., 2022).

### Enzymatic modification of antibiotics

Among the identified mechanisms underlying resistance, enzymatic modification of antibiotics (Table 1) represents a major contributor.Third-generation cephalosporin resistance in *E*. *coli* and *K. pneumoniae* is predominantly mediated by extended-spectrum β-lactamases (ESBLs), especially CTX-M, TEM, and SHV variants, which are often plasmid-associated and highly transmissible across bacteria (Castanheira et al., 2021). In contrast, resistance in *Serratia* sp., *Citrobacter* sp. (notably the *C. freundii* complex), and *Morganella* sp. is more frequently driven by chromosomally encoded, inducible AmpC β-lactamases, with plasmidic AmpC enzymes increasingly reported in both clinical and community isolates (Stankova et al., 2024; Thomson, 2010). These β-lactamase genes are commonly located on plasmids and transposable elements, facilitating horizontal gene transfer among Enterobacterales populations (Castanheira et al., 2021; Stankova et al., 2024).

Carbapenem resistance is predominantly mediated by *Carbapenemases*, including *bla*NDM, *bla*KPC, *bla*OXA-48, *bla*IMP, and *bla*VIM, which is widely distributed among Enterobacterales and *A. baumannii* (Reyes et al., 2020; Haider et al., 2022). In *A. baumannii*, acquired oxacillinases like *bla*OXA-23, *bla*OXA-24/40, and *bla*OXA-58 are prevalent and often plasmid-borne (Mat Ghani et al., 2025). Fluoroquinolone resistance in *Salmonella, Shigella,* and *Neisseria gonorrhoeae* is also associated with plasmid-mediated protection genes such as *qnr*B, *qnr*S, and the aminoglycoside acetyltransferase variant aac (6′)-Ib-cr, which modifies ciprofloxacin (Yanat, Rodríguez-Martínez, and Touati, 2017; Ruiz, 2019). in *Enterococcus faecium*, vancomycin resistance arises from acquiring *van*A and *van*B gene clusters, which alter the terminal amino acids of peptidoglycan precursors (Ramos et al., 2015). These genes are carried on conjugative plasmids and transposons, allowing rapid intra- and interspecies dissemination (Vo et al., 2024). Additionally, *Haemophilus influenzae* produces bla_TEM-1 and bla_ROB-1, β-lactamases that confer ampicillin resistance via mobile elements (Søndergaard et al., 2012).

### Modification of antibiotic targets

Modification of antibiotic targets is another prominent mechanism of antibiotic resistance (Table 1). In fluoroquinolone-resistant *Salmonella Typhi*, *Shigella* spp., and *N. gonorrhoeae*, point mutations in the quinolone resistance-determining regions (QRDRs) of *gyr*A*, gyr*B, *par*CE, and *par*C, reduce drug binding to DNA gyrase and topoisomerase IV (Nathania et al., 2022; Hall et al., 2019). Although these mutation are occur in the chromosome, the co-existence of plasmid-mediated *qnr* genes amplifies the resistance phenotype.

*Mycobacterium tuberculosis* bacterial resistance to rifampicin is driven by mutations in the *rpo*B gene. These mutations alter the bacterial RNA polymerase (β-subunit), preventing the effective antibiotic binding (Jagielski et al., 2018). In MRSA, the *mec*A gene produces a modified version of penicillin-binding protein (PBP2a) that reduced binding affinity to β-Lactam in antibiotics resulting to bacterial resistance (Lade and Kim, 2023). The mecA gene is located on a *Staphylococcal* cassette chromosome (SCCmec), a well-known mobile genetic element that facilitates its dissemination (Uehara, 2022).

In *Streptococcus pneumoniae* and Group A *Streptococcus*, resistance to macrolides is mediated either by the ribosomal methyltrenaserase (*erm*B) gene, which alters the drug’s target on the ribosome, or the efflux transporter *mef*A gene, which actively expels the antibiotic out of the cell and disseminate across streptococcal species (Schroeder and Stephens, 2016). In addition, both Group B *Streptococcus* and *S. pneumoniae* display resistance via mosaicpenicilin-binding proteins (PBPs) encoded by *pbp*1a, *pbp*2x, and *pbp*2b, typically acquired through homologous recombination with commensal *streptococci* to reduced β-Lactam susceptibility (Beres et al., 2022; Jensen et al., 2015).

### Biofilm formation

Biofilm formation provides both physical and physiological barriers to antimicrobial agents, significantly undermining antibiotic efficacy by limiting drug penetration and promoting stress-adapted bacterial phenotypes (Asma et al., 2022). Within biofils, bacteria exhibit altered growth rates and transcriptional profiles (Table 1), contributing to increased tolerance to antimicrobial compunds. *The K. pneumoniae*, *E. coli*, *Enterobacter* spp., and *Proteus* spp. utilize fimbrial adhesins for host cell attachment (e.g., fimH, mrkA) and regulatory factor such as luxS and pgaC to initiate and maintain biofilm formation (Dwyer et al., 2011; Murphy et al., 2013; Jagnow and Clegg, 2003). The *P. aeruginosa* forms robust biofilms via regulatory pathways involving *mex*R gene, which indirectly influences biofilm maturational stability and community persistence (Amudhan et al., 2012). In *A. baumannii*, biofilm development involves biofilm-associated protein (*bap),* the outer-membrane protein (*omp*A), and the *csu*A operon which collectively facilitate attachment to the surface and stability of biofilm (Jeong et al., 2024). Biofilm-associated genes such as *ica*A, *sig*B, and *sar*A in *S. aureus* (Pizauro et al., 2021) and the *csg*A gene in non-typhoidal *Salmonella* play critical roles in attachment, persistence, and tolerance to antimicrobial stress (Siddique et al., 2021).

Biofilms undermine the efficacy of antibiotics by limiting drug penetration through the extracellular polymeric matrix, and the bacteria within it develop a stress-adapted physiology that increases drug tolerance (Shenkutie et al., 2020; Azeem et al., 2025). Conseqeuntly, biofilm development often renders standard therapeutic doses ineffective, leading to the emergence of biofilm-related persistent infections associated with edical devices such as catheters and ventilators (Grooters et al., 2024). In addition, biofilms promote and increase horizontal gene transfer and enhance quorum-sensing activity. Quorum sensing also regulates the expression of resistance and virulence factors (Asma et al., 2022; Elshafiee et al., 2022).

These characteristics explain planktonic cells' vulnerability to inhibition, but sessile biofilm populations are resilient; agents that inhibit biofilm formation or interfere with quorum sensing are necessary. The possible approaches include phage-based solutions, natural compounds, and biofilm-disruptive therapies (Pejin et al., 2015; Eghbalpoor et al., 2024; Wickremasinghe et al., 2021).

### Horizontal gene transfer

The majority of clinically significant antimicrobial resistance genes particularly those encoding β-lactamases (e.g., bla_CTX-M, bla_NDM, and bla_KPC), aminoglycoside-modifying enzymes, efflux pumps (e.g., oqxAB, and qepA), and ribosomal protection proteins (e.g., ermB, and mefA), are located on mobile genetic materials such as plasmids, transposons, integrons, or bacteriophages and these mobile genetic materials enable the rapid dissemination of resistance traits across phylogenetically unrelated bacterial species through conjugation, transformation, and transduction (Partridge et al., 2018).

While β-lactamases represent a biochemical mechanism of antibiotic inactivation, their clinical impact is largely dictated by horizontal gene transfer, which facilitates the rapid interspecies dissemination of resistance genes through plasmids, integrons, and transposons (Tooke et al., 2019). Under experimental and hospital conditions, the vanA and vanB genes responsible for vancomycin resistance in *E. faecium* and are located on Tn1546-type transposons, transfer to *S. aureus* (Wardal et al., 2017). The mecA gene transmits via SCCmec elements in all *staphylococcal* species (Miragaia, 2018). Fluoroquinolone resistance genes of qnrS, qnrB, and aac (6′)-Ib-cr are plasmid-encoded and occur in both environmental and clinical isolates worldwide (Strahilevitz et al., 2009). Research shows that efflux pump genes oqxAB and qepA are located on mobile genetic elements, making them highly transferable. Often, these genes are physically linked on the same plasmid or transposon as other resistance genes, allowing a single transfer event to confer multi-drug resistance (Adekanmbi et al., 2022).

From a pharmacological standpoint, enzymatic drug inactivation and reduced permeability combined with efflux represent the greatest challenges, particularly in WHO-priority Gram-negative pathogens. These mechanisms directly limit intracellular drug exposure and compromise multiple antibiotic classes simultaneously. In contrast, target modification is more pathogen-specific, while biofilm formation primarily complicates chronic and device-associated infections rather than acute systemic disease.

## Critical evaluation and pharmacological implications

### ADMET and exposure constraints

A core reason many “promising” antibacterial candidates fail in late preclinical or early clinical development is unfavourable ADMET and exposure at the infection site, rather than lack of intrinsic antibacterial potency. For antibacterial pharmacology, the clinically relevant question is whether sufficient free (unbound) drug concentrations can be achieved and maintained at the site of infection (e.g., lung epithelial lining fluid, bone, abscess cavities, intracellular compartments, and biofilms) while preserving an acceptable safety margin. Modern PK/PD frameworks emphasize exposure targets, probability of target attainment, and the need to account for infection-site concentrations rather than plasma alone, particularly for difficult-to-penetrate compartments and high-inoculum infections (Onita et al., 2025; Ulrich and Hergenrother, 2025). In Gram-negative pathogens, outer-membrane permeability and active efflux frequently prevent adequate intracellular accumulation, creating a translational gap between biochemical activity and whole-cell efficacy and thereby raising ADMET-like constraints that are specific to antibacterials (Ulrich and Hergenrother, 2025; Farha et al., 2025). These limitations become even more decisive for non-traditional modalities (e.g., peptides, phages, and nucleic-acid–based antibacterials), where stability, clearance, immunogenicity, and biodistribution strongly determine effective exposure; therefore, ADMET profiling and compartment-focused PK/PD assessment should be considered early “go/no-go” filters for translational prioritisation (Olowu et al., 2025).

### Drug delivery and infection-site targeting

Successful antibacterial therapy depends not only on intrinsic activity but on whether effective concentrations can be delivered to the infection microenvironment. WHO-priority pathogens often persist in biofilms, necrotic foci, device matrices, or intracellular niches with poor drug penetration. Site-specific factors—diffusion barriers, altered pH/oxygen, high inoculum, and tolerance states—can reduce antimicrobial efficacy even when plasma levels are adequate (Grari et al., 2025). Delivery barriers vary across antimicrobial modalities and are key to pharmacological prioritisation. Conventional small molecules usually fail when adequate site exposure is unattainable or when toxicity prevents further dose escalation. By contrast, emerging strategies face additional platform-specific delivery liabilities: (i) bacteriophages show highly variable biodistribution and rapid clearance that complicate standardised dosing and sustained titres at target sites (Nguyen et al., 2026), and immune recognition can markedly accelerate phage removal from circulation (Nguyen et al., 2026). For antimicrobial peptides, clinical translation is frequently limited by proteolytic degradation and short systemic half-life; therefore, delivery systems (lipid/polymer/metal nanocarriers) and molecular modifications (e.g., cyclisation, terminal modifications) are increasingly treated as integral to pharmacological feasibility rather than optional formulation work (Gao et al., 2025). For CRISPR-based antimicrobials, the main bottleneck is achieving safe, efficient delivery into target bacteria in complex infections. Recent reviews highlight the need for improved vectors, such as phage or conjugation systems, and higher *in-vivo* delivery efficiency before clinical translation (Zhang et al., 2025). Consistent with this delivery challenge, early clinical development of CRISPR-enabled antibacterial strategies has primarily focused on bacteriophage-mediated delivery systems rather than standalone CRISPR therapeutics. For example, the CRISPR-Cas3–engineered bacteriophage cocktail LBP-EC01 has undergone human clinical evaluation for uncomplicated urinary tract infections, demonstrating feasibility while also highlighting the importance of scalable manufacturing, validated dosing strategies, and regulatory standardisation for broader implementation (Kim et al., 2024). For biofilm and intracellular infections, nano-enabled delivery is a promising strategy to enhance localisation, penetration, responsive release, and uptake into infected cells. However, key translational barriers remain, including scalability, target-site retention, and safety (Xie et al., 2025).

### Toxicity and therapeutic-index constraints

Toxicity is a decisive pharmacological constraint in antimicrobial therapy because the exposures required for efficacy must remain below thresholds that trigger organ injury or serious adverse events. In clinical practice, exposure–toxicity trade-offs are most visible where dosing must be individualized and monitored: for example, therapeutic drug monitoring (TDM) is used to control exposure and reduce nephrotoxicity risk for vancomycin in critically ill patients (Peng et al., 2025). Moreover, real-world data show that excess unbound β-lactam exposure can associate with nephrotoxicity and neurotoxicity, supporting a therapeutic-index framing rather than assuming that “more exposure is always better” (Temme et al., 2026).

For next-generation modalities, toxicity considerations extend beyond classic organ injury. Bacteriophage therapies are often described as having a favourable safety profile, yet translational frameworks emphasise immunological considerations and standardisation requirements that can affect both tolerability and dosing feasibility (Moon et al., 2025). For antimicrobial peptides, recent medicinal-chemistry work explicitly links stability-enhancing design (e.g., cyclization/stereochemical inversion) to improved proteolytic resistance while still requiring careful evaluation of haemolysis/cytotoxicity—illustrating that toxicity and stability must be optimised together for clinical translation (Wang et al., 2026). Finally, nanoparticle-enabled antimicrobials raise additional safety questions (e.g., biodistribution, organ accumulation, longer-term toxicity), and contemporary reviews consistently identify the limited depth of long-term safety datasets as a major translational barrier (Mouzakis et al., 2025).Accordingly, toxicity is considered a key determinant of the therapeutic index, influencing dose optimisation, regimen selection, and the clinical deployability of both conventional and emerging strategies against WHO-priority pathogens.

### Pharmacological barriers to antibiotic exposure

Across WHO-priority pathogens, the toughest pharmacological barrier is inadequate antibiotic exposure at the infection site. This is especially pronounced in Gram-negative bacteria, where enzymatic inactivation, low permeability, and active efflux can jointly undermine multiple drug classes (Lomovskaya and Bostian, 2006). These layered defenses explain why narrow, single-target therapies often underperform in complex infections, particularly biofilm-associated disease, where tolerance, physical protection, and altered metabolism diminish antibiotic efficacy (Ciofu and Tolker-Nielsen, 2019; Murray et al., 2022). Clinically realistic near-term progress therefore favours strategies that restore exposure and activity, such as β-lactamase inhibitor optimisation, rational combination therapy, and anti-biofilm adjuncts, while more technologically complex platforms remain constrained by delivery, safety, and regulatory challenges (Hawkey et al., 2018). Key unresolved challenges include translating mechanistic resistance insights into actionable pharmacokinetic–pharmacodynamic targets, clarifying how multiple resistance determinants interact *in vivo*, and reducing discrepancies between laboratory susceptibility models and real-world infections (Roberts et al., 2014). Future priorities should therefore focus on resistance-informed PK/PD optimisation, improved drug penetration into protected compartments such as biofilms and intracellular niches, and diagnostics capable of linking resistance mechanisms to therapy selection (Theuretzbacher et al., 2020; Murray et al., 2022). Beyond limiting immediate therapeutic efficacy, heterogeneous antibiotic penetration also creates conditions that favour resistance emergence during therapy. When drug penetration is spatially heterogeneous, such as within biofilms, poorly vascularised abscesses, or intracellular reservoirs, local antibiotic concentrations may fall into selection-permissive ranges even when systemic pharmacokinetic targets appear adequate. These exposure gradients allow tolerant subpopulations to persist and increase the probability that resistant mutants will be selected during ongoing treatment. The importance of exposure heterogeneity and compartment-restricted antibiotic activity has been emphasised in studies of Gram-negative permeability barriers, efflux-mediated intracellular exclusion, and biofilm-associated tolerance (Lomovskaya and Bostian, 2006; Ciofu and Tolker-Nielsen, 2019). These pharmacological realities help explain why laboratory susceptibility results do not always translate into clinical success, since real-world treatment failure often reflects inadequate infection-site exposure and pharmacokinetic variability rather than intrinsic drug inactivity (Roberts et al., 2014).

## Therapeutic challenges and limitations of conventional antibiotics

### Limitations of existing antibiotic classes

β-lactamases, including extended-spectrum β-lactamases (ESBLs), carbapenemases such as KPC, and NDM, can inactivate even the newly developed β-lactam antibiotics, including powerful combinations like ceftazidime-avibactam (β-lactam/β-lactamase advanced inhibitor combinations) (Muteeb et al., 2023). *P. aeruginosa* develops resistance by reducing drug uptake (via porin loss) and increasing drug expulsion (via efflux pumps) (Vergalli et al., 2020). Similarly, Fluoroquinolone resistance arises from mutations in the gyrA gene and acquired resistance genes (Rezaei et al., 2024). New treatments lag behind resistance from target modifications for vancomycin resistance in *Enterococcus faecium* and methicillin resistance in *S. aureus* (Li et al., 2021; Bakthavatchalam et al., 2022; D'Accolti et al., 2019). Biofilms of *A. baumannii* can require antibiotic concentrations hundreds of times higher to eradicate than their planktonic counterparts (Shenkutie et al., 2020). Conventional therapies are often inadequate for medical device-related infections because of combined resistance mechanisms (Abavisani et al., 2024).

### Failure of conventional drug discovery approaches

Few new antibiotics are truly novel, leaving them prone to existing resistance (Shenkutie et al., 2020). Soil bacteria are exhausted sources and drug modifications are frequently ineffective. Therefore, innovative approaches to antibiotic discovery and development remain inevitable (Martin, Alvarez-Alvarez, and Liras, 2022). Most preclinical drugs fail due to toxicity or poor absorption (Theuretzbacher et al., 2025). Ethical and practical challenges, such as strict inclusion requirements, limit clinical studies for multidrug-resistant (MDR) infections (Stewart et al., 2020). Single-target drugs fail against complex infections like biofilms. Persister cells survive antibiotics by entering a dormant state, a temporary non-genetic adaptation that allows them to endure treatment. Biofilms make therapy even more challenging as they provide a protected environment that favors the survival of these dormant cells. New strategies are now targeting dormant persister cells and resilient biofilms to overcome these treatment challenges (Stojowska-Swędrzyńska, et al., 2023). Due to funding limitations, novel techniques such as immuno-antibiotics are still not fully investigated (Fischbach and Walsh, 2009). Several antimicrobial approaches with strong experimental activity have failed in clinical translation due to predictable pharmacological and implementation barriers, including insufficient *in-vivo* exposure at infection sites, toxicity at effective doses, difficulty defining endpoints for non-traditional therapies, and challenges in scalable manufacturing and regulatory approval. Recognising these recurring obstacles is critical for prioritising development pathways and directing antimicrobial research toward clinically deployable strategies.

## Strategies and promising avenues for next-generation antimicrobial development

Given the rapidly expanding range of proposed antimicrobial innovations, a comparative overview of their current evidence base, translational readiness, and major development barriers is provided in Table 2 to contextualise their potential clinical roles against WHO-priority pathogens. Novel antimicrobial approaches are rapidly advancing to combat multidrug-resistant pathogens. New therapeutic targets, CRISPR-based tools, and host-directed therapies are increasingly feasible, offering improved immune enhancement with reduced disruption of the host microbiome (Kaufmann et al., 2018; Lin et al., 2021). Anti-virulence compounds such as quorum sensing inhibitors minimize bacterial fitness without inducing resistance (Naga et al., 2023; Starkey et al., 2014). AI-assisted multi-omics pipelines expedite drug discovery and target candidate identification (Ocana et al., 2025). Improvement in localization and safety of drugs by nanoparticle-based drug, delivery systems, and metabolically activated prodrugs (Campos et al., 2025). Engineered bacteriophages and synthetic biology can provide personalized pathogen-targeted therapies (Chinemerem Nwobodo et al., 2022; Ghaznavi et al., 2025). The combination of these approaches, in the context of diagnostics and genomic surveillance, is shaping what the future of antimicrobial therapy will look like (Kajumbula et al., 2024; Sherry et al., 2025). Natural-product–derived antimicrobials present additional pharmacological challenges, as variability in biological source, extraction procedure, and chemical composition can limit reproducibility and complicate dose standardisation. Furthermore, many natural antibacterial compounds demonstrate strong *in-vitro* activity but insufficient systemic bioavailability or infection-site exposure *in vivo*, necessitating rigorous chemical standardisation, pharmacokinetic evaluation, and formulation optimisation for clinical translation (Atanasov et al., 2021). Across these emerging strategies, translational success is critically dependent on pharmacological considerations, including ADMET behaviour, drug-delivery feasibility, toxicity profiles, resistance emergence during therapy, and dose optimization.

**TABLE 2:** Evidence strength, development barriers, and translational readiness of emerging antimicrobial strategies against WHO-priority pathogens^a^.

Strategy	Best-supported clinical use-case	Evidence strength	Translational readiness	Key barriers	Bottom-line author judgement	References
Resistance-modifying antibiotic combinations	Restore activity vs. MDR Gram-negative systemic infections	Human trials + clinical use	Near-term	resistance evolution; regimen optimisation	Most immediately deployable pharmacological approach	Castanheira et al., 2021; Philippon et al., 2022
Bacteriophage therapy	Refractory MDR infections (personalised/compassionate use)	Human cases + animal/mechanistic	Mid-term	regulation; PK/PD; scalable production	Promising for selected cases; needs standardisation	Schooley et al., 2017; Dedrick et al., 2019
Antimicrobial peptides (AMPs)	Local infections, device prevention, adjunct therapy	Strong *in-vitro* + animal; limited human	Mid-term (mainly local/adjunct)	toxicity; stability; PK	Likely first used topically or adjunctively	Mahlapuu et al., 2020; Chan et al., 2021
CRISPR antimicrobials	Target resistance genes or specific pathogens	Proof-of-concept + animal	Early-stage/high-risk	delivery; off-target; regulation	Highly innovative but delivery-limited	Bikard et al., 2014; Mayorga-Ramos et al., 2023
Anti-virulence/quorum-sensing inhibitors	Biofilm and chronic persistence infections	Mechanistic + animal	Early-to-mid	clinical endpoints; adjunct dependence	Best positioned as adjunct therapies	Fleitas Martínez et al. (2019)
AI-assisted antimicrobial discovery	Novel scaffold discovery and optimisation	Computational + early experimental	Early-to-mid	clinical validation; development timelines	Discovery accelerator, not development shortcut	Patel et al. (2020)
Natural-product-derived antimicrobials	Adjunct therapy or drug-scaffold sources	Mostly *in-vitro*; limited animal	Mid-term (mainly derivatives/adjuncts)	standardisation; bioavailability; toxicity	Strongest value in derivatives or adjunct use	Khare et al. (2021)

^a^ Strategies in Table 2 differ markedly in clinical readiness. Approaches restoring existing antibiotic activity offer the most realistic near-term impact, whereas CRISPR-based and AI-designed antimicrobials remain largely at the discovery stage. Phage therapy, antimicrobial peptides, and anti-virulence methods occupy an intermediate translational position. Overall, progress will depend not only on innovation but also on pharmacology, manufacturability, and clinical implementation.

### Novel antibiotic discovery: advancing beyond traditional molecules

The discovery and development of new antibiotics have become increasingly challenging in the face of the rapid emergence and global dissemination of antibiotic resistance (Ventola, 2015). Conventional methodologies to discover antibiotics usually revolve around finding more natural compounds or creating new molecules, but these options have been largely exhausted (Lewis, 2020). As bacteria grow more resistant to antibiotics, research should be more focused on new ways to find and create new antibiotics (Muteeb et al., 2023).

Synthetic biology could allow researchers to create novel molecular scaffolds by engineering microorganisms to make non-natural antibiotics (León-Buitimea et al., 2022). For instance, engineered *Escherichia coli* bacteria are currently employed in the production of new classes of antibiotics that inhibit bacterial membrane integrity and biofilm formation, who have never been targeted by traditional antibiotics before (Eghbalpoor et al., 2024).

Furthermore, scientists are also studying the bacteria that have not been studied before, such as bacterial ribosomes, transport systems, and metabolic pathways. These systems offer hope for the development of antibiotics which function differently. For example, compounds that inhibit nutrient transport or cause permeabilization of bacterial membranes are being studied as a new class of antibiotics (Chan et al., 2021). AI and ML are also critical for the prediction of the drug-like potential of these compounds, which can be further developed in the future.

### Overcoming resistance through repurposing and combination therapy

Drug repurpose has come to be considered a potential strategy for dealing with antibiotic resistance (Aggarwal et al., 2024). Scientists can therefore rapidly repurpose drugs that were initially developed for other therapeutic purposes to use against these drug-resistant pathogens (Aggarwal et al., 2024). Medicines designed to treat cancer, viral infections or even autoimmune diseases are being tested to see if they can increase the killing of bacteria by antibiotics (Chinemerem Nwobodo et al., 2022). For instance, the cytotoxic anti-cancer drug 5-fluorouracil that is not typically employed as an antibiotic, sensitized *Pseudomonas aeruginosa* to aminoglycoside tobramycin against resistant strains by interfering with bacterial metabolism and biofilm formation (Alibek et al., 2012).

Instead of repurposed drugs, combination therapy is another approach which is very promising in attempting to overcome bacterial resistance (Worthington and Melander, 2013). One example is the combination of a β-lactam antibiotic with a β-lactamase inhibitor, such as clavulanic acid, which protects the β-lactam antibiotic against resistance (Bush and Fisher, 2011). This therapy has been effective at treating infections, including those caused by *Haemophilus influenzae* and *Neisseria gonorrhoeae*. Other promising combinations are antibiotics with host-directed therapies, that is, drugs that modulate the immune system in order to make it more efficient in killing bacteria (even if the bacteria are resistant) (Taya, Teruyama, and Gojo, 2023).

One of the other potential combination therapies is using the antibiotic called meropenem, which is a broad-spectrum antibiotic to be used in combination with immunomodulatory drugs (Wickremasinghe et al., 2021). This strategy has proven to be effective in the treatment of multidrug-resistant strains of *P. aeruginosa* in clinical and preclinical settings and represents a new approach to combat challenging infectious diseases (Mengesha, 2025). Combination strategies are also employed to combat biofilm-related infections, in which bacteria create complex structures that protect them from antibiotics (Wickremasinghe et al., 2021).

### Phage therapy, antimicrobial peptides, and CRISPR-based approaches

As traditional antibiotics face increasing resistance rapidaly, alternative therapeutic approaches such as bacteriophage (phage) therapy, antimicrobial peptides (Lê et al., 2020), and CRISPR-based technologies are being explored as potential solutions (Table 3) (Lin, Koskella, and Lin, 2017). Phage therapy, the use of bacteriophages, viruses that infect bacteria, has attracted increasing interest recently amongst the scientific community. Because phages are very specific to the bacteria they target, they can be utilized to treat infections with a minimum impact on the host microbiota (Guo et al., 2024). Bacteriophages show strong potential against multidrug-resistant infections, highlight the need antimicrobial research and new strategies (Faruk et al., 2025). Unlike antibiotics, bacteriophages tend to have high specificity to a single species or even a specific stain. To avoid the resistaince, lytic bacteriophages can be used to a powerful tool eradicate AMR bacterial species in hospital and agricultural setting. Earlier studies have engineered phages to more effectively target biofilm forming bacteria (Kakkar et al., 2024). Researchers have effectively genetically modified phages, for instance, to improve their targeting power against biofilm-forming bacteria (Ghaznavi et al., 2025). However, widespread use requires resolving regulatory challenges and concerns about phage resistance (Pires et al., 2020). However, clinically, phage therapy remains constrained by complex pharmacokinetic behaviour, immune-mediated clearance, and challenges in dose standardisation, all of which directly affect therapeutic exposure and clinical efficacy. Unlike conventional antibiotics, phages replicate dynamically within the host and interact with bacterial density and host immune responses, meaning that effective dosing and treatment duration cannot be inferred solely from administered titre but must be evaluated within host–pathogen–immune system dynamics. Early clinical experience supports feasibility, including controlled human use in chronic otitis caused by antibiotic-resistant *P. aeruginosa* (Wright et al., 2009). However, larger clinical programmes have also exposed practical translational barriers: for example, the PhagoBurn phase 1/2 trial in burn wound infections demonstrated that formulation stability, achievable phage titre, and manufacturing consistency can significantly affect clinical evaluability, highlighting the need for GMP-compliant production, validated stability control, and standardised dosing frameworks before wider clinical adoption (Jault et al., 2019). Importantly, reduced clinical efficacy observed in parts of the study was linked not only to logistical formulation issues but also to insufficient active phage concentration at the infection site, illustrating how biological exposure constraints can directly influence clinical outcomes.

**TABLE 3:** Comparative overview of next-generation antimicrobial technologies.

Strategy	Mode of action	Advantages	Limitations	References
Phage Therapy	Infects and lyses specific bacteria	High specificity; minimal microbiome disruption; evolves with target bacteria	Regulatory hurdles; potential for phage resistance	Singha et al. (2024)
Antimicrobial Peptides	Disrupt bacterial membranes; interfere with intracellular processes	Broad-spectrum activity; low resistance potential	Stability and bioavailability challenges	Singha et al. (2024)
CRISPR-Based Approaches	Edits bacterial genome to remove or disable resistance genes	Precision targeting; restores antibiotic susceptibility	Delivery challenges; off-target concerns	Pacia et al. (2024)
Antivirulence Therapies	Inhibit bacterial virulence pathways (e.g., quorum sensing)	Reduced selective pressure for resistance; biofilm and toxin suppression	Does not directly kill bacteria; requires combination therapy	Bernabè et al. (2025)
Efflux Pump Inhibitors	Block multidrug efflux systems to increase intracellular antibiotic concentration	Can restore or enhance existing antibiotic efficacy	Specificity and toxicity challenges (note: not yet clinically approved)	Duffey et al. (2024)
Nanotechnology-Based Antimicrobials	Nanoparticles disrupt membranes, generate ROS, penetrate biofilms and deliver drugs	Multimodal killing; enhanced drug delivery; biofilm penetration	Toxicity concerns; regulatory/translation barriers	Campos et al. (2025)
Anti-Biofilm Agents	Disrupt biofilm matrix, signaling, or adhesion	Improves antibiotic penetration; addresses chronic/recurring infections	Often adjunctive; biofilm heterogeneity limits efficacy	Joshi and Patil (2025)
Host-Directed Therapies	Modulate host immunity to enhance pathogen clearance	Resistance-independent; broad applicability	Risk of immunopathology; complex host responses	Dominic Terkimbi et al. (2025)
Microbiome-Based/Bacteriotherapy	Restores healthy microbial communities to suppress pathogens	Reduces recurrence; microbiome-friendly	Individual variability; regulatory complexity	Romanova et al. (2025)

As a promosing alternative to conventional antibiotics, antimicrobial peptides (AMPs) exert their antimicrobial activity primarily by disrupt bacterial membranes and interfering with intracellular processes (Shriwastav et al., 2025). AMPs demonstrated a broad-spectrum alternative to antibiotics, especially against Gram-negative bacteria (Gani et al., 2025). However, AMP stability for clinical use continues to be a significant concern (Ali et al., 2025). From a pharmacological perspective, the clinical translation of antimicrobial peptides is limited by poor *in vivo* stability, low bioavailability, and rapid proteolytic degradation, which together constrain achievable dosing and therapeutic exposure (Theuretzbacher et al., 2020). In the coming years, antimicrobial peptide research is expected to shift from proof-of-concept discovery toward clinically optimized and indication-specific therapeutics. Advances in peptide engineering, including sequence optimization, cyclization, and incorporation of non-natural amino acids, will improve stability, selectivity, and safety profiles. Smart delivery systems (such as nanoparticle encapsulation, topical and localized formulations, and stimulus-responsive carriers) are anticipated to overcome bioavailability and toxicity limitations. In parallel, deeper mechanistic insights into host–pathogen interactions and immunomodulatory functions will enable rational combination therapies and precision applications, particularly in wound infections and biofilm-associated disease (Mahlapuu et al., 2020).

CRISPR-CAS is now being explored as innovative tool to combat antibiotic resistance (Ahmed, Kayode, et al., 2024). CRISPR technologies precisely target and edit bacterial genomes to disrupt resistance genes (Vivekanandan et al., 2025), restoring antibiotic sensitivity in resistant bacteria. CRISPR technology presents a precision-based method of fighting illnesses (Vivekanandan et al., 2025). CRISPR-based antibiotics show strong potential against antimicrobial resistance, though development remains early (Mayorga-Ramos et al., 2023). However, from a pharmacological perspective, CRISPR-based antimicrobial systems face major challenges related to safe and efficient *in vivo* delivery to bacterial targets, as well as the risk of off-target gene editing, both of which constrain dose control and clinical applicability (Bikard et al., 2014). Delivery inefficiency may substantially reduce the amount of active system reaching the infection site, thereby hindering attainment of effective exposure and often necessitating localized administration in early development. More broadly, limitations in delivery precision and target specificity influence biodistribution, bacterial targeting, and the overall therapeutic index, meaning that dosing strategies remain difficult to standardise and continue to depend on vector optimisation and site-specific delivery performance (Mayorga-Ramos et al., 2023).

## Clinical trials, real-world implementation, and implications for patient care

Over the past decade, several non-traditional antimicrobial strategies have entered early-phase clinical evaluation, with heterogeneous and often limited outcomes (Schooley et al., 2017). Phage therapy has shown encouraging results in compassionate-use cases and small clinical studies targeting multidrug-resistant *Mycobacterium abscessus, A*. *baumannii, P*. *aeruginosa*, and *S*. *aureus*, particularly in chronic, biofilm-associated, or device-related infections (Schooley et al., 2017; Dedrick et al., 2019). However, clinical translation of phage therapy has been constrained by substantial heterogeneity in trial design, the absence of standardized efficacy endpoints, and unresolved pharmacokinetic–pharmacodynamic complexities, which collectively limit progression toward large, randomized clinical trials (Palma and Qi, 2024).

In parallel, antimicrobial peptides have repeatedly demonstrated potent *in vitro* and preclinical activity but have failed to translate successfully in many clinical programs due to toxicity, instability, or insufficient systemic exposure (Mahlapuu et al., 2016). CRISPR-based antimicrobials remain largely at the preclinical stage, with current evidence deriving from *in vitro* and animal studies; significant challenges in delivery optimization, off-target effects, and regulatory frameworks continue to impede progression to advanced human clinical trial (Ahmed et al., 2024 a). In 2025, the U.S. Food and Drug Administration approved new antibacterial agents that modestly expand options for drug-resistant infections. Notably, gepotidacin (Blujepa), a first-in-class oral antibacterial, was approved for uncomplicated infections, and both gepotidacin and zoliflodacin (Nuzolvence) received approval for uncomplicated urogenital gonorrhea, addressing a long-standing therapeutic gap. These approvals were supported by phase III clinical trials demonstrating non-inferiority to standard regimens, although their clinical impact will depend on real-world implementation and antimicrobial stewardship (FDA, 2025).

Nanotechnology-driven and computational strategies, including *in silico* screening and fragment-based drug design, have enhanced target selectivity in early antimicrobial discovery. In parallel, multiple antibiotic alternatives, such as antimicrobial peptides, essential oils, quorum-sensing inhibitors, darobactins, bacteriophages, odilorhabdins, cannabinoids, and host- or metabolism-directed therapies, are being actively explored, alongside drug-repurposing efforts and novel chemical scaffolds. Despite promising preclinical and early clinical results, most remain at experimental or trial stages, with limited translation into widely approved antibacterial therapies (Niño-Vega et al., 2025). In general, we believe that no single approach will resolve the antibacterial drug crisis. Sustainable progress will require integrated strategies combining resistance-robust therapies, optimized use of legacy antibiotics, targeted metabolic interventions, and emerging technologies, supported by sustained investment, translational research, and strong interdisciplinary collaboration. In addition, important knowledge gaps remain regarding optimal pharmacokinetic targets for non-traditional antimicrobial therapies, standardised evaluation frameworks for biologic and precision-based interventions, and the long-term resistance dynamics associated with emerging treatment modalities. Contradictions also persist between laboratory efficacy studies and clinical outcomes, particularly where *in-vitro* susceptibility does not reliably predict therapeutic success in complex infections. Addressing these unresolved issues will be essential for improving translational predictability and guiding future antimicrobial research priorities (Stewart et al., 2020; Schooley et al., 2017). Over the next 5–10 years, clinical management of WHO-priority pathogens will likely improve incrementally rather than through disruptive breakthroughs. The largest near-term gains should come from optimising existing antibiotics via resistance-modifying combinations, faster diagnostics, and pharmacokinetic-guided dosing. Biologic options (e.g., phage therapy and antimicrobial peptides) may expand in niche uses such as chronic biofilm infections and compassionate-use cases, while advanced approaches like CRISPR-based antimicrobials will probably remain in early clinical evaluation due to delivery, regulatory, and manufacturing challenges.

### Targeting virulence factors and quorum sensing

New therapeutic strategies targeting virulence factors and quorum sensing to disarm bacterial development (Fleitas Martínez et al., 2019). Anti-virulence therapies disable bacterial disease mechanisms (Zhang et al., 2023). By weakening rather than killing bacteria, virulence factors may reduce infection severity while limiting resistance development. Further, quorum sensing is another promising target for antimicrobial therapy (Dahab, et al., 2023). Disruption of bacterial communication allows one to avoid the development of biofilms or the release of toxins, therefore greatly lowering the pathogenicity of bacteria (Leistikow et al., 2024). Apart from these developments, molecule inhibitors aiming at virulence factors are under development as well. For instance, it has been shown that inhibitors of the quorum sensing system of *Pseudomonas aeruginosa* greatly lower the pathogenicity of this infamous antibiotic-resistant bacteria (Rodríguez-Urretavizcaya, et al., 2024).

Although many next-generation antimicrobial strategies are under investigation, their readiness for clinical use differs markedly. Phage therapy and resistance-modifying combinations are closest to translation, whereas CRISPR-based approaches, nanotechnology, and AI-designed compounds remain largely preclinical. Anti-virulence strategies are best viewed as adjuncts rather than stand-alone therapies.

**FIGURE 3:**
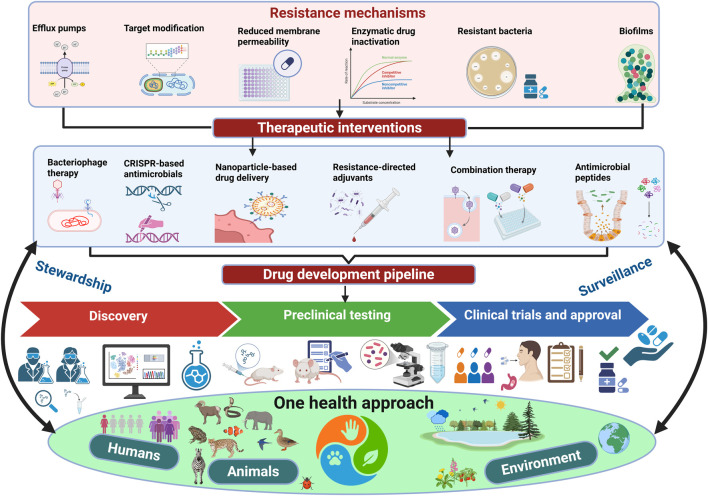
Integrated overview of antimicrobial resistance mechanisms, therapeutic strategies, translational pipeline, and One Health stewardship. Legend: The upper panel shows key bacterial resistance mechanisms that reduce antibiotic effectiveness. The middle panel presents therapeutic strategies developed to counter these mechanisms. The lower panel outlines the antimicrobial development pipeline from discovery to clinical use, framed within a One Health approach that links human, animal, and environmental sectors through stewardship and surveillance.

Despite strong experimental promise, the clinical translation of next-generation antimicrobials remains challenging due to difficulties in demonstrating *in vivo* efficacy, optimising dosing and pharmacokinetics, managing toxicity, and navigating regulatory and reimbursement barriers, underscoring the need to integrate clinical pharmacology, regulatory science, and stewardship early in development (Theuretzbacher et al., 2020). Additional translational barriers include regulatory uncertainty for non-traditional antimicrobials, particularly phage and CRISPR-based therapies, which do not fit easily within existing approval frameworks designed for conventional small-molecule antibiotics. Unlike conventional antibiotics, biologically adaptive or personalised antimicrobials such as bacteriophages challenge traditional regulatory paradigms because product composition may change between patients, complicating standardised clinical trial design, batch certification, and long-term pharmacovigilance (Pires et al., 2020). Manufacturing scalability also remains a major challenge, as personalised or pathogen-specific approaches complicate standardisation, quality control, and large-scale production. For example, personalised phage cocktails often require rapid pathogen-specific isolation, amplification, and purification under GMP conditions, a workflow substantially more complex than standardised small-molecule antibiotic manufacturing (Kortright et al., 2019). Furthermore, cost-effectiveness, reimbursement models, and equitable access represent critical constraints, especially in low- and middle-income settings, underscoring the need for regulatory adaptation, sustainable manufacturing strategies, and economic incentives to support clinical adoption (Outterson et al., 2016). Even when technically effective, adoption of advanced biologic antimicrobials may be constrained by reimbursement uncertainty, specialised laboratory infrastructure requirements, and limited accessibility in routine hospital settings, factors that have historically slowed uptake of several novel antibacterial platforms. Figure 3 shows an integrated overview of antimicrobial resistance mechanisms, corresponding therapeutic strategies, the translational development pipeline, and the role of One Health stewardship and surveillance.

### Computational and AI-assisted approaches in antimicrobial discovery

Advances in computational biology, artificial intelligence (AI), structural biology, and bioinformatics are increasingly supporting antibiotic discovery by enabling systematic identification of therapeutic targets and optimisation of candidate molecules. Machine learning approaches allow high-throughput screening of large chemical libraries and prediction of antimicrobial activity based on structural–activity relationships, pharmacological profiles, and resistance trends (Rahman et al., 2022; Ali, Ahmed, and Aslam, 2023; Anahtar, Yang, and Kanjilal, 2021). Deep-learning models can recognise complex molecular patterns and guide structural optimisation of compounds to improve antibacterial potency and pharmacological characteristics, while genomic and multi-omics analyses help prioritise targets by identifying pathogen vulnerabilities and biosynthetic gene clusters associated with antibiotic production (Louwen et al., 2023; Chernov et al., 2019). In addition, predictive modelling can help assess resistance trends by analysing genome sequences and resistance profiles (Orcales et al., 2024).

Structural biology provides the molecular framework necessary for rational antibiotic design by revealing the architecture of bacterial targets at near-atomic resolution. Techniques such as X-ray crystallography and cryo-electron microscopy have enabled detailed visualisation of ribosomes, membrane complexes, and enzymatic active sites, allowing direct identification of binding pockets, allosteric regions, and conformational changes relevant to drug interaction (Kühlbrandt, 2012; Razi, Britton, and Ortega, 2017). These insights are particularly valuable for understanding resistance mechanisms at the molecular level—for example, structural studies of ribosomal protection proteins such as ABCF ATPases have informed efforts to design antibiotics capable of bypassing these defences (Sharkey, Edwards, and O’Neill, 2016; Takada, Sugimoto, and Oshima, 2025).

Building on structural information, *in silico* drug design enables virtual screening of extensive chemical libraries against defined targets using molecular docking platforms such as AutoDock, PyRx, and GOLD, allowing estimation of binding affinity and interaction stability before laboratory validation (Agamah et al., 2020; Dahab et al., 2023; Sulieman et al., 2024). Molecular dynamics simulations further refine these predictions by modelling ligand–target interactions under dynamic physiological conditions, while integrated machine-learning approaches can evaluate absorption, distribution, metabolism, excretion, and toxicity (ADMET) properties during early optimisation stages (Wang et al., 2015; Venkataraman et al., 2025). Modern structural-prediction systems, including AlphaFold-based tools, have expanded the pool of potentially druggable bacterial proteins and opened new avenues for target identification and inhibitor design.

Bioinformatics complements these approaches by enabling large-scale genomic surveillance and resistance mapping. Next-generation sequencing permits rapid identification of resistance genes, mobile elements, and mutation patterns associated with antimicrobial failure, while comparative genomic analysis reveals polymorphisms, insertions, and gene-transfer events driving resistance evolution (Khaledi et al., 2016; Madden et al., 2021). Databases such as CARD, ResFinder, and AMRFinderPlus support systematic annotation of resistance determinants (Feldgarden et al., 2021; Florensa et al., 2022; McArthur et al., 2013), and metagenomic sequencing allows discovery of novel resistance genes and regulatory pathways in uncultivable microbial communities (Abramova, Karkman, and Bengtsson-Palme, 2024; Maia et al., 2024). These computational frameworks therefore provide essential insight into the emergence, evolution, and dissemination of antimicrobial resistance.

Despite these technological advances, translation into clinically deployable antibiotics remains challenging. Several promising strategies have stalled during development because experimental efficacy alone does not guarantee therapeutic success. Antimicrobial peptides frequently encounter stability, toxicity, and pharmacokinetic limitations *in vivo*, CRISPR-based antimicrobial systems face substantial delivery and regulatory challenges, and nanotechnology-based approaches raise concerns regarding biodistribution, safety, and scalable manufacturing. Although AI-assisted discovery has markedly improved target identification and candidate prioritisation, most computationally identified compounds remain at the preclinical stage. These limitations highlight that successful antibiotic innovation depends not only on computational discovery but also on effective delivery systems, validated safety profiles, and realistic clinical implementation pathways. Key computational and AI-assisted approaches currently used to accelerate antimicrobial discovery, compound optimisation, and target identification are summarised in Table 4.

**TABLE 4:** Computational and AI-assisted tools supporting antimicrobial discovery, optimisation, and target identification.

Tool/Approach	Application area	Representative examples	Key advantages for antimicrobial discovery
Machine learning models	Antimicrobial activity prediction; resistance pattern analysis	Deep-learning–based hit identification from chemical libraries	Accelerates candidate screening, handles large-scale datasets, enables identification of novel antimicrobial scaffolds and resistance-associated patterns
Structure–activity modelling	Rational drug design based on molecular feature analysis	QSAR modelling and regression-based predictive models	Supports scaffold optimisation, predicts potency and toxicity profiles, and prioritises compounds for experimental validation
Molecular docking simulations	Virtual screening and binding affinity estimation	AutoDock, PyRx, GOLD	Enables rapid structure-based screening, predicts ligand–target interactions, and reduces experimental workload by focusing wet-lab validation on high-probability candidates
Molecular dynamics simulations	Analysis of molecular interactions and stability over time	GROMACS, NAMD	Evaluates binding stability, conformational flexibility, and dynamic protein–ligand behaviour, improving confidence in docking predictions
AlphaFold2/protein structure prediction	Identification of structural targets including previously unresolved proteins	Structural models of unknown bacterial proteins	Expands the druggable target space, enables structure-informed inhibitor design, and supports downstream virtual screening workflows

### Natural products: rediscovering forgotten antimicrobials

The growing interest in natural products as first-line interventions against infections reflects the resistance mechanisms and therapeutic gaps discussed earlier in this review. Many phytochemicals, microbial metabolites, and marine-derived compounds target pathways used by WHO-priority pathogens to evade treatment by different means (Abdallah et al., 2023; Bharathi and Lee, 2024). By acting on multiple bacterial systems rather than a single target, natural products offer a clear advantage against multidrug-resistant organisms and represent a complementary strategy for next-generation antimicrobial development.

Natural products and plant-derived antimicrobials are once again being considered as vital sources to combat antibiotic resistance (Angelini, 2024). These natural agents take many forms and work in many ways that usually do not exist among man-made drugs (Álvarez-Martínez et al., 2025). Natural chemicals from plants, or phytochemicals, can damage the bacteria’s outer layers, block their vital enzymes, and reduce these bugs’ ability to cause disease (Dalir Abdolahinia et al., 2023). A number of these plant-derived compounds have already proven to work well against bacteria that are otherwise difficult to kill with common antibiotics.

We have thousands of years of experience with plants, and many of them are used for treatment in traditional medicine, which provides a useful guide as to which ones might be effective from natural sources. Improved methods of laboratory testing and scientific tools have helped researchers test much more quickly and accurately how well these natural compounds work. By returning to nature and advancing their understanding of these powerful agents, scientists are developing new ways to attack drug-resistant infections that can be safer and eco-friendlier (Mungwari et al., 2025). Although, from a clinical pharmacology perspective, natural products face persistent challenges related to standardisation of extracts, batch-to-batch reproducibility, and often poor or variable bioavailability, all of which complicate dose optimisation and regulatory translation (Atanasov et al., 2021).

### Phytochemicals with antibacterial potential

Phytochemicals, chemicals found in plants, have attracted much attention as potential substitutes or complements to traditional antibacterial drugs (Barbieri et al., 2017). These bioactive molecules, which include alkaloids, flavonoids, terpenoids, tannins, saponins, and phenolic acids, have multiple modes of antibacterial action (Table 5). They can either act on bacterial membranes, nucleic acid synthesis, quorum sensing, or virulence factor expression (Khare et al., 2021). Structurally diverse and evolutionarily well conserved, peptides have become promising agents for tackling the worsening problem of AMR (Murugaiyan et al., 2022).

**TABLE 5:** Representative antibacterial phytochemicals: sources, mechanisms of action, and bacterial targets.

Phytochemical	Plant source	Antibacterial target	Mechanism of action	References
Berberine	*Berberis* sp	*E. coli, S. aureus*, MDR strains	Inhibits DNA replication; disrupts membrane; inhibits efflux pumps	Kosalec et al. (2022)
Allicin	*Allium sativum* (garlic)	Broad-spectrum, including biofilm-forming bacteria	Modifies thiol enzymes; disrupts metabolism and quorum sensing	Bhatwalkar et al. (2021)
Curcumin	*Curcuma longa* (turmeric)	*P. aeruginosa*, *S. aureus*	Disrupts membranes; anti-quorum sensing	Zheng et al. (2020)
Epigallocatechin gallate (EGCG)	*Camellia sinensis* (green tea)	Gram-positive and Gram-negative pathogens	Inhibits efflux pumps; synergises with antibiotics	Capasso et al. (2025)
Quercetin	*Allium cepa* (onion), *Capsicum annuum* (chili pepper)	*S. aureus*, *E. coli*	Inhibits DNA gyrase; disrupts membrane integrity	Nguyen and Bhattacharya (2022)
Resveratrol	*Vitis vinifera* (grape skin)	*Listeria monocytogenes*, *Salmonella* spp.	Inhibits ATP synthase; interferes with quorum sensing	Ma et al. (2018)
Thymol	*Thymus vulgaris* (thyme)	*Bacillus subtilis*, *E. coli*	Disrupts membrane permeability; inhibits enzyme activity	Nagoor Meeran et al. (2017)
Cinnamaldehyde	*Cinnamomum verum* (cinnamon)	*S. aureus*, *P. aeruginosa*	Inhibits cell division; disrupts membrane and biofilm formation	Shu et al. (2024)
Eugenol	*Syzygium aromaticum* (clove)	*S. aureus, E. coli, P. aeruginosa,* MDR strains	Cytoplasmic membrane disruption; leakage of cellular contents; antibiofilm activity	Marchese et al. (2017)
Carvacrol	*Origanum* and *Thymus* plants	Erythromycin-resistant Group A *Streptococci*	Damage of bacterial membrane	Magi et al. (2015)
p-Cymene	*Thymus, Cuminum, Origanum* spp.	*E. coli, S. aureus*	Membrane swelling; potentiates phenolic monoterpenes	Marchese et al. (2017)
Baicalein	*Scutellaria baicalensis*	MRSA, *S. aureus*	Inhibition of biofilm formation; interference with quorum sensing and virulence factor expression	Mu et al. (2025)
Glycyrrhetinic acid	*Glycyrrhiza glabra* (Liquorice root)	*S. aureus*, MRSA	Disruption of bacterial growth, perturbation of metabolic gene expression and transport processes	Oyama et al. (2016)
Rosmarinic acid	*Rosmarinus officinalis*	*S. aureus*, MRSA	Suppresses expression of microbial surface components recognising adhesive matrix molecules	Ekambaram et al. (2016)
Saponins	*Quillaja, Yucca*, legumes	Gram-positive bacteria	Membrane destabilisation and increased permeability	Jubair et al., 2021; Khan et al., 2018
Tannic acid	*Oak galls*, tea, berries	*E. coli, S. aureus*	Membrane/cell envelope disruption; metal-ion chelation; interference with metabolic proteins/peptidoglycan synthesis; anti-biofilm activity	Villanueva et al. (2023)

The broad-spectrum activity versus MDR pathogens such as *E. coli, S*. *aureus, P*. *aeruginosa, and K*. *pneumoniae* has been demonstrated for several phytochemicals. For instance, berberine, an isoquinoline alkaloid extracted from Berberis spp., inhibits bacteria through mechanisms of action against DNA replication and membrane permeability (Wojtyczka et al., 2014). It also blocks efflux pumps, re-establishing the antibiotic action of tetracycline and ciprofloxacin (Morita et al., 2016). Allicin, a sulphur-containing compound derived from garlic (*Allium sativum*), is capable of killing a broad range of microorganisms by affecting thiol-containing enzymes and interfering with the microbial metabolism. The capacity to repress biofilm development and quorum sensing makes it an important agent in the combating of non-resolving infections (Borlinghaus, et al., 2014).

More importantly, the synergistic effect of phytochemicals with antibiotics is turning out to be a powerful approach to defeat the resistance (Barbieri et al., 2017). It has been reported that berberine in combination with β-lactams or macrolides and allicin in combination with aminoglycosides could significantly decrease minimum inhibitory concentrations (MICs) of antibiotics for resistant strains (Wojtyczka et al., 2014; Borlinghaus, et al., 2014). These synergistic analyses can block resistance mechanisms such as those involving β-lactamase, efflux pump, or biofilm maturation, and can help to recover the activity of antibiotics. In addition to direct antibacterial activity, many phytochemicals also influence the host immune response or function as anti-quorum sensing agents, which means that they can inhibit the activity of quorum sensing in bacteria that are virulent but do not have a selective microbiological effect (Alhazmi et al., 2021; Asfour, 2018).

Moreover, several natural products have emerged as reference molecules in modern antimicrobial discovery due to their potent activity against multidrug-resistant (MDR) pathogens. Teixobactin, discovered from *Eleftheria terrae*, targets the highly conserved cell wall precursors lipid II and lipid III and exhibits strong activity against MRSA, VRE, and Clostridioides species, with no resistance observed under experimental conditions to date (Dashtbani-Roozbehani and Brown, 2021). Daptomycin, a cyclic lipopeptide produced by *Streptomyces roseosporus*, disrupts bacterial membrane potential and is clinically effective against MRSA, VRE, and biofilm-associated Gram-positive infections, although its activity can vary depending on biofilm maturity and environmental conditions (Ling et al., 2015; Haeusser and Margolin, 2016). In addition, platensimycin and platencin, isolated from *Streptomyces* platensis, inhibit bacterial fatty acid biosynthesis through FabF and FabH, respectively, and display potent activity against MRSA and other Gram-positive pathogens (Palanichamy and Kaliappan, 2010; Shang et al., 2015).

### Ethnopharmacology

**FIGURE 4:**
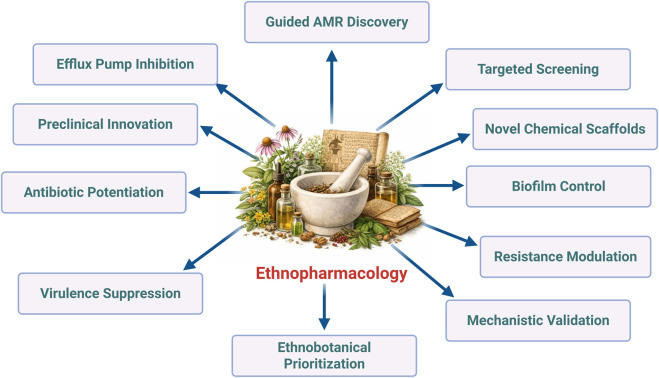
Ethnopharmacology as a Strategic Framework for the Discovery and Control of WHO-Priority Pathogens. Legend: Conceptual framework positioning ethnopharmacology as a strategic driver of antimicrobial discovery and resistance control against WHO-priority pathogens. Ethnobotanical prioritization guides targeted screening and identification of novel bioactive scaffolds, while mechanistic validation and preclinical innovation translate traditional knowledge into evidence-based interventions. Through this integrative approach, plant-derived compounds contribute to efflux inhibition, antibiotic potentiation, biofilm control, virulence suppression, and resistance modulation, supporting guided AMR discovery and complementing conventional drug-development pipelines.

Ethnopharmacology represents an additional source of antimicrobial leads, as it provides a structured way to translate traditional medical knowledge into testable antimicrobial hypotheses, thereby reducing the search space compared with purely random screening (Figure 4). Although ethnopharmacological knowledge still requires rigorous validation and safety assessment, this rationale has been widely discussed in the context of drug discovery from medicinal plants and remains relevant for antimicrobial resistance, where new chemical matter, adjuvants, and anti-virulence strategies are urgently needed (Fabricant and Farnsworth, 2001).

From an AMR perspective, ethnopharmacology is valuable not only for identifying direct-growth inhibitors but also for finding multi-target agents and resistance-modifying adjuvants, as mentioned earlier in this study. These activity classes are particularly relevant in the context of WHO-priority bacterial pathogens because plant-derived compounds can act as antibiotic adjuvants that help restore susceptibility, while anti-virulence approaches may attenuate pathogenicity without directly targeting bacterial viability and therefore may impose less selective pressure than conventional bactericidal monotherapy. Accordingly, ethnopharmacology can be framed in this review as a practical route to (i) new chemical scaffolds, (ii) antibiotic potentiators, and (iii) anti-virulence leads aligned with next-generation antimicrobial strategies (WHO, 2024). A robust ethnopharmacology pipeline typically includes: (1) careful documentation of traditional indications and preparation routes; (2) botanical authentication and voucher specimen deposition; (3) extraction that reflects traditional use (alongside standardised extracts for reproducibility); (4) bioassay-guided fractionation linked to clinically meaningful endpoints (MIC/MBC, biofilm inhibition, synergy/potentiation, anti-virulence readouts); (5) chemical characterisation (LC–MS/GC–MS/NMR); (6) mechanism-orientated assays (Figure 1); and (7) early safety/quality gates (cytotoxicity, haemolysis, selectivity index, stability, batch-to-batch consistency). This “field-to-function” framework clarifies how traditional medicine knowledge can be converted into evidence suitable for modern antimicrobial development (Fabricant and Farnsworth, 2001; Patwardhan, 2005). Ethnopharmacology provides a practical route to convert complex natural mixtures into lead-like compounds for drug discovery. This method is all about finding bioactive compounds and making natural product–derived or semi-synthetic analogues that are stronger, more selective, better at getting into the body, and safer. Standardised multi-component extracts remain acceptable when their activity is reproducible, mechanistically justified, and quality-controlled. In this context, ethnopharmacology fits well within modern natural-product-based drug discovery rather than serving only as a descriptive account of traditional use (Atanasov et al., 2021).

However, because genetic resources and associated traditional knowledge support ethnopharmacology, researchers must clearly address ethical and legal compliance with access and benefit-sharing frameworks, including the Nagoya Protocol. This is necessary for non-commercial research, international collaboration, and downstream development In parallel, quality and safety challenges (such as adulteration, misidentification, batch variability, and herb–drug interactions) should also be explicitly considered when dealing with ethnopharmacological prescription for AMR control (Colella et al., 2023).

Ethnopharmacology should not be considered a direct substitute for antibiotics. Its main value lies in identifying lead compounds and resistance-modifying adjuvants that can support combination strategies, rather than serving as stand-alone treatments.

### Marine and soil microbial metabolites as a source of new antibiotics

Marine environments are increasingly recognized as a rich source of novel antimicrobial agents (Bharathi and Lee, 2024). The great biodiversity of marine organisms, including bacteria, fungi, and algae, may have a broad spectrum of biologically active compounds with significant antimicrobial properties (Bhatnagar and Kim, 2010). Many of these compounds are structurally unique because of the extreme conditions (high salinities, pressures, and low temperatures) that exist in marine environments where these organisms live (Dalmaso et al., 2015). These distinctive features render them valuable for innovative antibiotic research.

For instance, *Pseudomonas fluorescens* and *Bacillus subtilis*, environmental bacteria commonly isolated from soil and aquatic habitats, have been reported to produce antibacterial metabolites (Jia et al., 2023). Marine algae, such as the red algae species, also were reported to be able to produce bioactive agents with antibacterial activity, and they represent attractive reservoirs of novel antimicrobials (Balkrishna et al., 2025; Shannon and Abu-Ghannam, 2016). Marine-derived antibiotics such as abyssomicin C inhibit folate synthesis and show potent activity against MDR Gram-positive organisms (Sadaka et al., 2018).

Likewise, soil microorganisms, and more specifically Actinobacteria, have already been known for their ability to produce antibiotics (Hu et al., 2020). Recent genomic approaches have discovered additional antibiotic biosynthetic pathways in soil microbes that can now be explored in the search for new antibiotics from this resource-rich habitat. Many more antibiotics, including streptomycin (one of the original antibiotics), would follow the discovery (Hu et al., 2020).

### Synergistic effects between natural and synthetic compounds

The combined use of natural and synthetic antimicrobials is a promising strategy on the horizon in addressing resistance. This approach combines the synergistic actions of plant-based molecules and common antibiotics to increase the efficacy of the latter, in particular with respect to MDR pathogens (Elmaidomy et al., 2022). Specific phytochemicals derived from medicinal plants, notably curcumin from *Curcuma longa* and cinnamaldehyde from *Cinnamomum verum*, have been shown to disrupt bacterial cytoplasmic membranes, increasing membrane permeability and thereby facilitating enhanced intracellular penetration of conventional antibiotics (Elmaidomy et al., 2022). This action, which breaks down membranes, makes bacteria that are resistant to drugs more likely to respond to drugs that they were resistant to before.

As previously mentioned, efflux pumps are one of the major resistance mechanisms. Phytochemicals such as quercetin, epigallocatechin gallate (EGCG), and berberine are reported to inhibit the pumps and increase the intracellular levels of antibiotics, which can regain the antibiotic potency (Dos Santos et al., 2021; Duda-Madej et al., 2025). This strategy is not limited to increasing the efficacy of present antibiotics but also to diminishing the dosage, which may lead to lower toxicity and fewer side effects.

Additionally, by blocking biofilm formation (the primary obstacle to antibiotic penetration and the cause of chronic infections), natural compounds may be effective. Biofilm protects bacterial consortia with a dense extracellular matrix, which may be up to 1,000-fold more resistant to antibiotics (Azeem et al., 2025; Grooters et al., 2024). Flavonoids, including quercetin and resveratrol, are described as intercepting quorum sensing pathways and inhibiting biofilm maturation, rendering bacteria more accessible to antibiotics (Pejin et al., 2015).

Likewise, the combined application of both natural and synthetic antifungal drugs is also interesting for avoiding the development of resistance (Dantas et al., 2025). These combinations have an additive effect by attacking several bacterial pathways at a time so that the chance of the resistance mutation is less. Furthermore, multiple natural products demonstrate anti-inflammatory and immunomodulatory effects that may contribute to host defence (Moudgil and Venkatesha, 2022; Sai Priya, Ramalingam, and Suresh Babu, 2024).

### The role of traditional medicine in antimicrobial discovery

Conventional medicine has formed the basis of healthcare in cultures throughout the world for hundreds of years, providing a plethora of plant-derived drugs with which to treat infectious diseases (Fabricant and Farnsworth, 2001). People are increasingly embracing these traditional practices, many rooted in traditional plant-based indigenous knowledge systems, as promising avenues for new antimicrobial drug discovery (Rates, 2001).

*Andrographis paniculata*, commonly used in Ayurvedic and Southeast Asian traditional medicine, is a case in point (Intharuksa et al., 2022). Extracts of *Andrographis paniculata* (especially methanol- and chloroform-based) have shown inhibitory effects against both Gram-positive (e.g., *Staphylococcus aureus, B. subtilis*) and Gram-negative bacteria (e.g., *Escherichia coli, Salmonella typhi*) (Dafur et al., 2024). The active principle, andrographolide, is widely recognised for its anti-inflammatory and antimicrobial properties, rendering it an interesting lead candidate for future pharmaceutical development (Burgos et al., 2020; Li et al., 2022).

Likewise, *Echinacea purpurea*, a valuable plant in Native American medicine, has been found to have an antimicrobial effect against respiratory pathogens such as *Streptococcus pneumoniae* and *Haemophilus influenzae* (Ahmadi, 2024; Vimalanathan et al., 2025). The bioactive components of Echinacea, like alkamides, caffeic acid derivatives, and polysaccharides, account for its immunostimulatory and antimicrobial activities (Ahmadi, 2024).

There are several benefits for the incorporation of traditional medicinal knowledge in modern drug discovery pipelines. It minimises the hunting grounds for bioactives, enhances therapeutic outcomes, and preserves the cultural history of native populations. Rapid developments in phytochemistry, genomics, and bioassay-guided fractionation tools have now made it possible to identify and purify active ingredients in traditional remedies with unprecedented accuracy.

### Future perspectives: one health, stewardship, and surveillance

#### Integrating one health approaches to combat antibiotic resistance

In response to the global threat of antimicrobial resistance (AMR), a One Health framework is essential because selection and transmission occur across interconnected human, animal, food, and environmental systems. In the veterinary sector, antimicrobials used for therapy, metaphylaxis, and prophylaxis in livestock and companion animals create sustained selection pressure that amplifies multidrug-resistant bacteria and mobile resistance genes (Van Boeckel et al., 2015; Marshall and Levy, 2011). These determinants can disseminate across sectors through direct animal–human contact, occupational exposure, and the food production chain, while policy restrictions have demonstrated measurable reductions in resistance prevalence (Tang et al., 2017).

Agricultural practices further connect AMR with environmental ecology: manure application, contaminated irrigation water, and agricultural runoff introduce antibiotics and resistance genes into soils and crops, shaping environmental resistomes and facilitating horizontal gene transfer (Berendonk et al., 2015). Importantly, environmental compartments are not passive sinks but active reservoirs and mixing grounds where resistance genes persist and circulate, particularly in wastewater treatment systems and surface waters influenced by anthropogenic discharge (Rizzo et al., 2013). Co-selective pressures such as heavy metals and biocides further enrich resistance determinants even in the absence of high antibiotic concentrations (Seiler and Berendonk, 2012). Accordingly, effective control of WHO-priority pathogens requires integrated cross-sector strategies that reduce antimicrobial selection pressure in clinical, veterinary, agricultural, and environmental domains.

Antibiotic use in both humans and animals is monitored, and antibiotics are prescribed only when needed to prevent resistance, for example. In addition, the treatments of wastewater are imperative to lessen the environmental burden of antibiotics and resistant bacteria (Grehs et al., 2021; Talat et al., 2025).

#### Strengthening surveillance and stewardship programmes

Antibiotic stewardship programmes play a key role in antibiotic use control in human and veterinary medicine. These programmes help to ensure that antibiotics are prescribed only when necessary and that the right antibiotic is used for the right infection (Ha, Haste, and Gluckstein, 2019).

So, it has been proven to work in successful stewardship countries (like Sweden and Denmark). If we implement such approaches worldwide, we will have made an important step to reduce resistance and maintain the effect of antibiotic drugs in the current usage (Eriksen et al., 2021; Björkman et al., 2022).

In addition to drug discovery, AI is increasingly being applied to the monitoring and prediction of AMR trends. AI-assisted surveillance systems can combine genomic, clinical, and epidemiological data to find new resistance hotspots, predict how diseases will spread, and help with stewardship interventions. Such tools hold promises for strengthening global surveillance networks and enabling proactive responses to resistance threats (Singh and Sodhi, 2025). Interestingly, as mentioned before, recent advances in AI have enabled large language model–based tools to support evidence synthesis in AMR research. In this context, LLMzCor, an LLM-driven classification framework, has been used to automatically screen and categorise large volumes of literature related to the WHO priority antibiotic-resistant bacteria. This approach offers a scalable complement to conventional reviews and facilitates high-level assessment of research trends following the WHO’s critical bacteria list (Robledo Almonacid et al., 2025).

On the other side, effective control and stewardship of WHO-priority antimicrobial-resistant pathogens requires a genuinely integrated One Health approach that recognises the interconnected roles of human health, animal production, and the environment in resistance emergence and spread (WHO, 2022; United Nations Environment Program, 2023). Antimicrobial use in veterinary medicine and agriculture remains a major driver of selection pressure, enabling resistant bacteria and resistance genes to circulate through food systems and occupational exposure (Murray et al., 2022). Environmental compartments, particularly wastewater and surface waters, function as critical reservoirs and mixing grounds for resistance determinants, facilitating cross-species and cross-sector transmission (Abdallah, 2025). From a One Health perspective, antimicrobial stewardship must therefore extend beyond clinical settings to include veterinary prescribing, agricultural regulation, and environmental surveillance, supported by coordinated cross-sector monitoring systems to reduce overall selection pressure and preserve antimicrobial effectiveness (WHO, 2022). Operationally, antimicrobial stewardship must be implemented as a coordinated cross-sectoral system rather than a purely clinical intervention. Within human healthcare, stewardship programmes emphasise rational prescribing, appropriate antibiotic selection, and surveillance-guided treatment optimisation to reduce unnecessary antimicrobial exposure (Ha et al., 2019). However, effective containment of resistance requires extending stewardship principles to veterinary medicine, agriculture, and environmental management, recognising that antimicrobial use in animal production and food systems contributes substantially to selection pressure and resistance dissemination across human populations (WHO, 2022; Murray et al., 2022; United Nations Environment Program 2023).

Beyond clinical and veterinary settings, environmental stewardship plays a critical role, as antibiotic residues, resistant bacteria, and resistance genes can persist and circulate through wastewater systems and environmental reservoirs, reinforcing the need for monitoring and mitigation strategies targeting pharmaceutical discharge and waste management (Grehs et al., 2021; Talat et al., 2025).

Countries that have implemented coordinated stewardship policies integrating surveillance, regulatory control, and antimicrobial-use monitoring across sectors have demonstrated measurable reductions in inappropriate antibiotic use and resistance trends, highlighting the effectiveness of structured national stewardship frameworks (Eriksen et al., 2021; Björkman et al., 2022). Collectively, these observations reinforce that sustainable control of WHO-priority pathogens requires stewardship systems that simultaneously address clinical prescribing, veterinary antimicrobial use, agricultural practices, and environmental dissemination pathways.

#### Collaborative efforts in advancing next-generation antibiotics

International coordination is key to new antibiotic development. Public-private partnerships (PPPs) may overcome some of the financial and logistical obstacles that currently impede antibiotic development. More investment in research and development and collaboration in sharing data and resources will speed up the discovery of new antibiotics.

Another issue is access, because new antibiotics must be accessible to more people, including those in low-income countries. Programmes such as the Global Antibiotic Research and Development Partnership (GARDP) help make new antibiotics accessible to the people who need them most, especially in areas hardest hit by resistant infections (Ahmed, et al., 2024 b).

### Limitations

This review has several limitations that should be acknowledged. First, it is a narrative review and therefore does not follow PRISMA guidelines or include a formal quantitative synthesis or risk-of-bias assessment. While this approach allows integration of evidence from different fields, it may introduce some degree of selection bias. Second, the literature search was limited to English-language publications published between 2010 and 2024, which may have excluded relevant studies published in other languages or earlier work. Although multiple databases and authoritative reports were consulted, the review is not exhaustive, and some very recent findings may not yet be included.

Third, the included studies differ widely in terms of pathogens, experimental models, study design, and outcome measures. This heterogeneity limits direct comparison across studies. In addition, publication bias may have influenced the available evidence, as studies reporting positive findings are more likely to be published. Finally, many of the emerging therapeutic strategies discussed in this review are supported mainly by preclinical or early-stage evidence. Their clinical effectiveness and safety remain to be confirmed through well-designed clinical trials.

Finally, unlike many reviews that catalogue resistance mechanisms or list emerging technologies separately, this review adopts a mechanism-guided pharmacological perspective. Major resistance pathways, permeability restriction, enzymatic inactivation, efflux, target modification, and biofilm formation, are interpreted as determinants of drug exposure, therapeutic index, and clinical failure. By linking molecular resistance to pharmacokinetic–pharmacodynamic constraints and infection-site exposure barriers, the review provides a translational bridge between microbiology and clinical drug development.

Emerging strategies, including resistance-modifying combinations, nanoparticle delivery systems, antimicrobial peptides, CRISPR-based tools, and phage therapy, are assessed not only for experimental activity but for feasibility, safety, delivery challenges, and regulatory readiness, distinguishing near-term options from longer-horizon innovations. These elements are integrated within a One Health framework, recognising that resistance evolves across interconnected human, animal, and environmental systems.

## Conclusion

Antimicrobial resistance in WHO-priority pathogens represents a pharmacological and clinical systems challenge rather than a single drug-discovery problem. Evidence reviewed here indicates that the most realistic short-term clinical gains will arise from strategies that restore or optimise the activity of existing antibiotics, including resistance-modifying adjuvants, rational combination therapy, anti-biofilm interventions, and improved pharmacokinetic-guided dosing. These approaches offer the highest probability of near-term patient benefit because they build on validated therapeutic frameworks and established safety knowledge.

In addition, progress in antimicrobial pharmacology is unlikely to be driven primarily by the rare emergence of wholly new antibiotic classes. Instead, decisive advances will come from aligning drug development with resistance biology: refining existing scaffolds to evade dominant mechanisms, deploying PK/PD-optimised combination regimens that constrain evolutionary escape, adopting adaptive clinical trial designs centred on contemporary multidrug-resistant pathogens, and embedding these strategies within implementation frameworks that operate effectively under real clinical and stewardship constraints.

More advanced technologies, including bacteriophage therapy, antimicrobial peptides, host-directed therapies, and AI-assisted discovery pipelines, show important potential but remain variably constrained by delivery limitations, manufacturing scalability, regulatory complexity, and incomplete human exposure data. Their clinical adoption is therefore likely to expand first in specialised or refractory infection settings rather than routine frontline therapy.

From a research perspective, priority should shift toward mechanism-guided therapeutic design, clinically relevant infection models, and early integration of pharmacokinetic, safety, and manufacturability criteria into antimicrobial development pipelines. For clinicians, optimal deployment of existing agents through stewardship, combination strategies, and infection-specific optimisation remains the most immediately impactful intervention. For policymakers, sustained investment in surveillance infrastructure, regulatory adaptation for biologic antimicrobials, and incentives supporting antibiotic innovation will be essential to maintain an effective therapeutic arsenal.

Finally, successful control of WHO-priority pathogens will depend on coordinated integration of molecular microbiology, clinical pharmacology, translational science, and public-health policy rather than reliance on single breakthrough technologies.
