# Supplementary material for: The WHO priority list of antibiotic-resistant bacteria: challenges and opportunities for next-generation antimicrobial development
Source: Front Pharmacol. 2026 Apr 10;17:1699987. doi: 10.3389/fphar.2026.1699987 (PMC13106162; doi:10.3389/fphar.2026.1699987)
Supplement: Supplementary file 1 [file Table1.pdf]

Supplementary Material

Table S1. Mechanisms and genetic determinants of defining antibiotic resistance in WHO priority pathogens.

**Pathogen**	**Group**	**Defining Antibiotic**	**Permeability Alterations**	**Efflux Pump Genes**	**Modification of Antibiotic Genes**	**Modification of Target Genes**	**Biofilm/Regulatory Genes**	**Genetic Context**	**References**
Klebsiella pneumoniae, 3rd-gen cephalosporin-resistant	Critical	3rd-gen cephalosporins	Mutations in ompk36, ompk37 genes	–	*blaSHV, blaTEM, blaCTX-M*	–	*fimA, fimH, rmpA,rmpA2*	IncFII plasmids	Quan et al. 2023; Sahoo et al. 2024
Klebsiella pneumoniae, carbapenem-resistant	Critical	Carbapenems	*ompK35/36* loss	*acrAB, oqxAB*,	*blaKPC, blaNDM*, *blaOXA*	–	*mrkA, mrkD, mrkH, luxS, pgaA*	Tn4401	Tsai et al. 2011; Fang et al. 2021
Escherichia coli, 3rd-gen cephalosporin-resistant	Critical	3rd-gen cephalosporins	ompC, ompF	acrAB, yhiv, acrD, acrF, emrE, mdfA, emrAB, emrD, tehA	*blaCTX-M blaTEM bla-SHV*	–	*marA*	IncI1 plasmids	Sionov and Steinberg 2022; Hayer et al. 2020
Escherichia coli, carbapenem-resistant	Critical	Carbapenems	*ompF/C* porin loss	*acrA, acrB, tolC*, mexA, mexB	*blaKPC blaNDM, blaOXA blaIMP blaTEM*	–	-	IncFI,and IncX3 plasmids	Li, Ye, et al. 2021; Chen, Li, et al. 2024
Morganella spp., 3rd-gen cephalosporin-resistant	Critical	3rd-gen cephalosporins	–	emrAB	*blaAmpC, blaCTX-M*	–	*ampr, hdcT1,hdcT2, hisRS*	-	Park et al. 2020,Zaric et al. 2021

Enterobacter spp., carbapenem-resistant	Critical	Carbapenems	*ompF/C* ompK35, ompK36 loss	*acrAB*-tolC, mdfA	*blaKPC, blaNDM*	–	*csgD, bcsA,rpoS*	Tn3 transposons	Lê et al. 2020; Devanga et al. 2020
Enterobacter spp., 3rd-gen cephalosporin-resistant	Critical	3rd-gen cephalosporins	*ompF/C* ompK35, ompK36 ompX loss	*acrAB-tolC*	*blaCTX-M, blaAmpC*	–	*marA,SoxS, Rob, RamA, RarA*	Plasmids (IncHI2)	Ferrand et al. 2020
Citrobacter spp., 3rd-gen cephalosporin-resistant	Critical	3rd-gen cephalosporins	–	–	*blaCMY, blaCTX-M*	–	*ompX*, *bssS*	IncA/C2 plasmids	Philippon et al. 2022; Moser et al. 2021
Proteus spp., 3rd-gen cephalosporin-resistant	Critical	3rd-gen cephalosporins	–	–	*blaCTX-M, blaTEM*	–	*ureR*, *zapA*	IS26 cassettes	Karpenko et al. 2024; Sun et al. 2020
Serratia spp., 3rd-gen cephalosporin-resistant	Critical	3rd-gen cephalosporins	ompC loss	*SdeXY-HasF*	*blaCTX-M bla-SHV, bla-TEM*	–	*flhD*, *phoPQ*	Intl1	Sionov and Steinberg 2022; Liang et al. 2023
Acinetobacter baumannii, carbapenem-resistant	Critical	Carbapenems	*CarO/OmpA* loss	*adeABC*, *adeIJK*, *adeFGH* mexAB-oprM	*blaOXA, blaNDM*	*PBPs* mutations	*bap*, *CsuE*, *abaI*	ISAba1	Xu et al., 2019; Shenkutie et al. 2020
Mycobacterium tuberculosis, rifampicin-resistant	Critical	Rifampicin	ponA2	mmr, mmpL7, Rv1258c, p55, efpA	–	*Mutations in rpoB*	relA pks16 Rv2224c	IS6110	Richards et al. 2019
Salmonella Typhi, fluoroquinolone-resistant	High	Fluoroquinolones	–	*acrAB-tolC*	*qnrS*	*gyrA* (S83F), *parC* (S80I)	*ramA*, *marA*	Plasmids (IncHI2)	Sionov and Steinberg 2022; Chen et al. 2016
Shigella spp., fluoroquinolone-resistant	High	Fluoroquinolones	–	–	*qnrA, qnrS*	*gyrA* (S83L), *parC* (S80I)	–	ISCR1	Zhang et al. 2019
Enterococcus faecium, vancomycin-resistant	High	Vancomycin	–	–	–	*vanA, vanB*	esp gene	Tn1546	O'Toole et al. 2023; Freitas et al. 2011

Pseudomonas aeruginosa, carbapenem-resistant	High	Carbapenems	*oprD* loss	*mexAB-oprM*,	*blaVIM, blaIMP*	*ampC* mutations	*mexR*	Class 1 integrons	Sionov and Steinberg 2022; Elshafiee et al. 2022
Non-typhoidal *Salmonella*, fluoroquinolone-resistant	High	Fluoroquinolones	ompC/F	*acrAB-tolC*	*qnrS, qnrB*	*gyrA* (S83F), *parC* (S80I)	*csgA*	Plasmids (IncHI2)	Siddique et al. 2021; Jiang et al. 2023
Neisseria gonorrhoeae, fluoroquinolone-resistant	High	Fluoroquinolones	–	*mtrCDE*	–	*gyrA parC*	–	Chromosomal	Hall et al. 2019; Kivata et al. 2019
Staphylococcus aureus (MRSA)	High	Methicillin	–	*norA*, *mepA*	–	*mecA* (SCCmec)	*icaA*, *sigB*, *sarA*	SCCmec cassettes	Pizauro et al. 2021; Rezende-Júnior et al. 2020
Neisseria gonorrhoeae, 3rd-gen cephalosporin-resistant	High	3rd-gen cephalosporins	–	*mtrCDE*	*blaTEM*, *penA*	*ponA* mutations	–	Genetic Island	Shaskolskiy et al. 2022
Group A *Streptococci*, macrolide-resistant	Medium	Macrolides	–	*mefA*, *msrD*	–	*ermB, ermTR*	–	Tn917	Chuang et al. 2015; Wu et al. 2024
Streptococcus pneumoniae, macrolide-resistant	Medium	Macrolides	–	*mefA/E*, *msrD*	–	*ermB, ermTR*	–	Mega element	Mosleh et al. 2014; Schroeder and Stephens 2016
Haemophilus influenzae, ampicillin-resistant	Medium	Ampicillin	–	AcrAB-TolC	*blaTEM, blaROB*	*ftsI* mutations	Pili, hif	ICEHin1056	Maher and Hassan 2023; Sionov and Steinberg 2022

Legend: This table compiles representative molecular mechanisms and associated genetic determinants underlying clinically relevant antibiotic resistance in WHO-priority bacterial pathogens. Entries summarize commonly reported resistance genes, regulatory elements, and genomic contexts linked to reduced drug susceptibility, including permeability alterations, efflux systems, enzymatic inactivation, target modification, and biofilm-associated factors. The listed determinants reflect defining or frequently documented resistance features reported in the primary literature and major surveillance studies rather than exhaustive genomic inventories. References correspond to representative mechanistic or epidemiological studies cited in the main manuscript. This supplementary dataset supports the mechanistic patterns summarized in Table 1 of the article.

References

Chen, R., Li, C., Ge, H., Qiao, J., Fang, L., Liu, C., Gou, J., and Guo, X. (2024). Difference analysis and characteristics of incompatibility group plasmid replicons in gram-negative bacteria with different antimicrobial phenotypes in Henan, China. *BMC Microbiol.* 24:64.

Chen, W., Fang, T., Zhou, X., Zhang, D., Shi, X., and Shi, C. (2016). IncHI2 plasmids are predominant in antibiotic-resistant *Salmonella* isolates. *Front. Microbiol.* 7:1566.

Chuang, P. K., Wang, S. M., Lin, H. C., Cho, Y. H., Ma, Y. J., Ho, T. S., Shen, C. F., and Liu, C. C. (2015). The trend of macrolide resistance and emm types of group A streptococci from children at a medical center in southern Taiwan. *J. Microbiol. Immunol. Infect.* 48, 160–167.

Devanga Ragupathi, N. K., Muthuirulandi Sethuvel, D. P., Triplicane Dwarakanathan, H., Murugan, D., Umashankar, Y., Monk, P. N., Karunakaran, E., and Veeraraghavan, B. (2020). The influence of biofilms on carbapenem susceptibility and patient outcome in device-associated *Klebsiella pneumoniae* infections. *Front. Microbiol.* 11:591679.

Elshafiee, E. A., Khalefa, H. S., Al-Atfeehy, N. M., Amer, F., Hamza, D. A., and Ahmed, Z. S. (2022). Biofilms and efflux pump regulatory gene (mexR) in multidrug-resistant *Pseudomonas aeruginosa*. *Vet. World* 15, 2425–2431.

Fang, R., Liu, H., Zhang, X., Dong, G., Li, J., Tian, X., Wu, Z., Zhou, J., Cao, J., and Zhou, T. (2021). Difference in biofilm formation between carbapenem-resistant and carbapenem-sensitive *Klebsiella pneumoniae*. *Microb. Pathog.* 152:104743.

Ferrand, A., Vergalli, J., Pagès, J. M., and Davin-Regli, A. (2020). An intertwined network of regulation controls membrane permeability in Enterobacteriaceae. *Microorganisms* 8:833.

Freitas, A. R., Coque, T. M., Novais, C., Hammerum, A. M., Lester, C. H., Zervos, M. J., Donabedian, S., Jensen, L. B., Francia, M. V., Baquero, F., and Peixe, L. (2011). Human and swine hosts share vancomycin-resistant *Enterococcus faecium* clonal clusters. *J. Clin. Microbiol.* 49, 925–931.

Hall, C. L., Harrison, M. A., Pond, M. J., Chow, C., Harding-Esch, E. M., and Sadiq, S. T. (2019). Genotypic determinants of resistance in *Neisseria gonorrhoeae*. *Sex Health* 16, 479–487.

Hayer, S. S., Lim, S., Hong, S., Elnekave, E., Johnson, T., Rovira, A., Vannucci, F., Clayton, J. B., Perez, A., and Alvarez, J. (2020). Genetic determinants of resistance in *Escherichia coli*. *mSphere* 5.

Jiang, Y., Wang, Z. Y., Li, Q. C., Lu, M. J., Wu, H., Mei, C. Y., Shen, P. C., Jiao, X., and Wang, J. (2023). Characterization of XDR *Salmonella enterica* isolates. *Microbiol. Spectr.* 11:e0321922.

Karpenko, A., Shelenkov, A., Petrova, L., Gusarov, V., Zamyatin, M., Mikhaylova, Y., and Akimkin, V. (2024). Multidrug-resistant *Proteus mirabilis* clones. *Heliyon* 10:e40821.

Kivata, M. W., Mbuchi, M., Eyase, F. L., Bulimo, W. D., Kyanya, C. K., Oundo, V., Muriithi, S. W., Andagalu, B., Mbinda, W. M., Soge, O. O., McClelland, R. S., Sang, W., and Mancuso, J. D. (2019). gyrA and parC mutations in *Neisseria gonorrhoeae*. *BMC Microbiol.* 19:76.

Li, F., Ye, K., Li, X., Ye, L., Guo, L., Wang, L., and Yang, J. (2021). Genetic characterization of carbapenem-resistant *Escherichia coli*. *BMC Microbiol.* 21:248.

Liang, Z., Shen, J., Liu, J., Sun, X., Yang, Y., Lv, Y., Zheng, J., Mou, X., Li, H., Ding, X., and Yang, F. (2023). *Serratia marcescens* in bovine mastitis. *Infect. Drug Resist.* 16, 2727–2735.

Maher, C., and Hassan, K. A. (2023). The Gram-negative permeability barrier. *mBio* 14:e0120523.

Mosleh, M. N., Gharibi, M., Alikhani, M. Y., Saidijam, M., and Vakhshiteh, F. (2014). Macrolide resistance genes in *Streptococcus pneumoniae*. *Iran J. Basic Med. Sci.* 17, 595–599.

Moser, A. I., Keller, P. M., Campos-Madueno, E. I., Poirel, L., Nordmann, P., and Endimiani, A. (2021). Carbapenemase-producing Enterobacteriaceae. *Pathog. Immun.* 6, 119–134.

O'Toole, R. F., Leong, K. W. C., Cumming, V., and Van Hal, S. J. (2023). Vancomycin-resistant *Enterococcus faecium*. *Res. Microbiol.* 174:104046.

Park, S. Y., Lee, K., Cho, Y., Lim, S. R., Kwon, H., Han, J. E., and Kim, J. H. (2020). Cephalosporin-resistant *Morganella morganii*. *Animals* 10.

Philippon, A., Arlet, G., Labia, R., and Iorga, B. I. (2022). Class C β-lactamases. *Clin. Microbiol. Rev.* 35:e00150-21.

Pizauro, L. J. L., de Almeida, C. C., Silva, S. R., MacInnes, J. I., Kropinski, A. M., Zafalon, L. F., de Avila, F. A., and Varani, A. M. (2021). Mastitis-related staphylococci genomics. *Sci. Rep.* 11:17392.

Quan, J., Hu, H., Zhang, H., Meng, Y., Liao, W., Zhou, J., Han, X., Shi, Q., Zhao, D., Wang, Q., Jiang, Y., and Yu, Y. (2023). Plasmid-mediated resistance transfer. *Microbiol. Spectr.* 11:e03554-22.

Rezende-Júnior, L. M., Andrade, L. M. S., Leal, A., Mesquita, A. B. S., Santos, A., Neto, J. S. L., Siqueira-Júnior, J. P., Nogueira, C. E. S., Kaatz, G. W., Coutinho, H. D. M., Martins, N., da Rocha, C. Q., and Barreto, H. M. (2020). Chalcones as efflux pump inhibitors. *Antibiotics* 9.

Richards, J. P., Cai, W., Zill, N. A., Zhang, W., and Ojha, A. K. (2019). Biofilm adaptation in *Mycobacterium tuberculosis*. *Antimicrob. Agents Chemother.* 63.

Sahoo, S., Routray, S. P., Mohanty, J. N., Pattnaik, A., Nayak, D. S. K., Shah, S., Das, J., Subudhi, E., and Swarnkar, T. (2024). XDR *Klebsiella pneumoniae* ST15. *Gene Rep.* 36:101978.

Schroeder, M. R., and Stephens, D. S. (2016). Macrolide resistance in *Streptococcus pneumoniae*. *Front. Cell Infect. Microbiol.* 6:98.

Shaskolskiy, B., Kandinov, I., Dementieva, E., and Gryadunov, D. (2022). Antibiotic resistance in *Neisseria gonorrhoeae*. *Microorganisms* 10.

Shenkutie, A. M., Yao, M. Z., Siu, G. K. H., Wong, B. K. C., and Leung, P. H. M. (2020). Biofilm-induced resistance in *Acinetobacter baumannii*. *Antibiotics* 9:817.

Siddique, A., Azim, S., Ali, A., Andleeb, S., Ahsan, A., Imran, M., and Rahman, A. (2021). Biofilm-forming *Salmonella enterica*. *Antibiotics* 10.

Sionov, R. V., and Steinberg, D. (2022). Targeting quorum sensing and biofilms. *Microorganisms* 10:1239.

Sun, Y., Wen, S., Zhao, L., Xia, Q., Pan, Y., Liu, H., Wei, C., Chen, H., Ge, J., and Wang, H. (2020). Biofilm and resistance in *Proteus mirabilis*. *BMC Vet. Res.* 16:176.

Tsai, Y. K., Fung, C. P., Lin, J. C., Chen, J. H., Chang, F. Y., Chen, T. L., and Siu, L. K. (2011). Outer membrane porins in *Klebsiella pneumoniae*. *Antimicrob. Agents Chemother.* 55, 1485–1493.

Wu, X., Alibayov, B., Xiang, X., Lattar, S. M., Sakai, F., Medders, A. A., Antezana, B. S., Keller, L. E., Vidal, A. G. J., Tzeng, Y. L., Robinson, D. A., Stephens, D. S., Yu, Y., and Vidal, J. E. (2024). Macrolide resistance acquisition in *Streptococcus pneumoniae*. *Drug Resist. Updat.* 77:101138.

Xu, C. F., Bilya, S. R., and Xu, W. (2019). adeABC efflux gene in *Acinetobacter baumannii*. *New Microbes New Infect.* 30:100549.

Zaric, R. Z., Jankovic, S., Zaric, M., Milosavljevic, M., Stojadinovic, M., and Pejcic, A. (2021). *Morganella morganii* infections. *Indian J. Med. Microbiol.* 39, 404–412.

Zhang, W. X., Chen, H. Y., Tu, L. H., Xi, M. F., Chen, M., and Zhang, J. (2019). Fluoroquinolone resistance in *Shigella*. *Microb. Drug Resist.* 25, 212–218.
